# Structural Studies based on two Lysine Dioxygenases with Distinct Regioselectivity Brings Insights Into Enzyme Specificity within the Clavaminate Synthase-Like Family

**DOI:** 10.1038/s41598-018-34795-9

**Published:** 2018-11-08

**Authors:** Karine Bastard, Tatiana Isabet, Enrico A. Stura, Pierre Legrand, Anne Zaparucha

**Affiliations:** 1 Génomique Métabolique, Genoscope, Institut François Jacob, CEA, CNRS, Univ Evry, Université Paris-Saclay, 91057 Evry, France; 2 Synchrotron SOLEIL, L’Orme des Merisiers, Saint-Aubin, BP 48, 91192 Gif-sur-Yvette, France; 3 CEA, Institut des Sciences du Vivant Frédéric Joliot, Service d’Ingénierie Moléculaire des Protéines (SIMOPRO), Université Paris-Saclay, Gif-sur-Yvette, 91190 France

## Abstract

Iron(II)/α-ketoacid-dependent oxygenases (αKAOs) are enzymes that catalyze the oxidation of unactivated C-H bonds, mainly through hydroxylation. Among these, those that are active towards amino-acids and their derivatives are grouped in the Clavaminate Synthase Like (CSL) family. CSL enzymes exhibit high regio- and stereoselectivities with strict substrate specificity. This study reports the structural elucidation of two new regiodivergent members, KDO1 and KDO5, active towards lysine, and the structural and computational analysis of the whole family through modelling and classification of active sites. The structures of KDO1 and KDO5 in complex with their ligands show that one exact position in the active site controls the regioselectivity of the reaction. Our results suggest that the substrate specificity and high stereoselectivity typical of this family is linked to a lid that closes up in order to form a sub-pocket around the side chain of the substrate. This dynamic lid is found throughout the family with varying sequence and length and is associated with a conserved stable dimeric interface. Results from this study could be a starting-point for exploring the functional diversity of the CSL family and direct *in vitro* screening in the search for new enzymatic activities.

## Introduction

Enzymes derived from microorganisms are particularly appealing for the design of chemical processes that are environmentally friendly and sustainable^1^. Enzymes catalyze interconversions of many functional groups with well-defined chemo-, regio- and stereoselectivities, resulting in high atom economy and reduced waste during synthesis. Over the past 25 years, the tremendous progress in molecular biology, bioinformatics and process optimization have lowered the biotechnological barriers that were limiting the industrial use of enzymes^2–4^. Much research has looked for novel enzymes able to catalyze challenging reactions such as the functionalization of non-activated (i.e. not alpha to C-C or C-heteroatom double bonds) C-H bonds. Such reactions still resist most efforts made by organic chemists. However, enzymes that can catalyze the formation of various C-heteroatom bonds have been found^5,6^. We have focused our work on the iron(II)/α-KetoAcid-dependent Oxygenases (αKAOs), a mechanistically diverse superfamily of enzymes that use dioxygen as the oxidant to catalyze several reactions via C-H bond activation, mostly hydroxylation^7–9^. These enzymes operate with three substrates, dioxygen, α-ketoglutarate (α-KG) and a primary substrate. One oxygen atom is transferred to a primary substrate to give an oxidized product, and the second oxygen is given to the α-KG, which upon oxidation, forms succinate and CO_2_^10,11,12^.

Many αKAOs are tailoring enzymes involved in secondary metabolite biosynthesis. Such enzymes, often found in bacteria, have been reported to hydroxylate the side chains of free amino acids and derivatives, or tether peptides in non-ribosomal peptide biosynthesis^13^. Since hydroxylated amino acids contain several stereogenic centers, they are valuable chiral building blocks for fine chemical synthesis, which justifies the intensive effort to search for enzymes that can perform such reactions^14,15^. Recently, by using a genomic approach that combines sequence comparison and protein-domain sharing, we have discovered enzymes that exclusively catalyze the stereoselective hydroxylation of amino acids with basic side chains (arginine, ornithine and lysine)^16^. These enzymes, that act specifically on L-lysine (named KDO for K DiOxygenases), are highly stereoselective with different regioselectivities. One of these, KDO1, catalyzes the formation of (3*S*)-3-hydroxy-L-lysine, while five others, KDO2 to KDO6, promote the formation of (4*R*)-4-hydroxy-L-lysine.

To understand the observed C3/C4-regioselectivity, and as most of the αKAOs active towards amino acids and their derivatives are C3 regioselective, we performed a structural and computational analysis of the enzyme active sites. We extended our work to the family of the αKAOs superfamily to which our KDOs belong, to identify which structural elements are responsible for the substrate specificity and regioselectivity of the hydroxylation reaction of amino acids and derivatives. We based our analysis on the structures that we solved by X-ray crystallography of two of the regiodivergent αKAOs active towards L-lysine, namely KDO1 from *Catenulispora acidiphila*, that catalyzes the C-3 hydroxylation, and KDO5, from *Flavobacterium* sp., that catalyzes the C-4 hydroxylation.

## Results and Discussion

### Overall description of KDO1 and KDO5 structures

The crystal structures of KDO1 (C-3 regioselective) and KDO5 (C-4 regioselective) were both determined in several states. The structure of the apo form of KDO1 was determined without iron in the active site, lost during purification, while KDO5 retained the iron throughout. Moreover, the various complexes, with L-lysine, α-KG and the products of the reaction, succinate and hydroxy-L-lysine, were obtained by soaking crystals. The resolution of the structures obtained ranged from 2.6 to 1.9 Å (Tables S1 and S2). Four molecules characterize the asymmetric unit for both enzymes (Fig. S1A,B). The dimeric assembly (Fig. S1C) is similar for both KDO1 and KDO5. The surface buried in the interfaces of A-B and C-D chains varies, from 1380 Å^2^ for KDO1 to 1490 Å^2^ for KDO5. Two dimers assemble to give four molecules in the asymmetric unit, both for KDO1 and KDO5. The area buried in such interfaces is only 462 Å^2^ for KDO1 (B-D) but 627 Å^2^ for KDO5 (B-C). Solution experiments show a dimer as the native assembly for KDO1, and a tetramer for KDO5 (data not shown), analysis of which are reported Tables S1 and S2. Like VioC, a jelly-roll motif characterizes the core of the protein formed by two β-sheets composed of five (ABGDI) and three (EHF) strands each (Fig. S2)^17^. The outside is covered by eight α-helices (α1-α8). Three helices, α2, α3 and the shorter C-terminal α8 form the dimer interfaces A-B and C-D in KDO1 and KDO5 while in VioC, the dimer is also crystallographic, formed by A-A’ .

### Flexible loops

KDO5 can be distinguished structurally from all other structurally related αKAOs due to the protruding flexible loop (Gly320-Arg334, Fig. S1D). Other loops are poorly ordered in electron density and have not been interpreted in the PDB deposition (Table S3).

**Figure 1:**
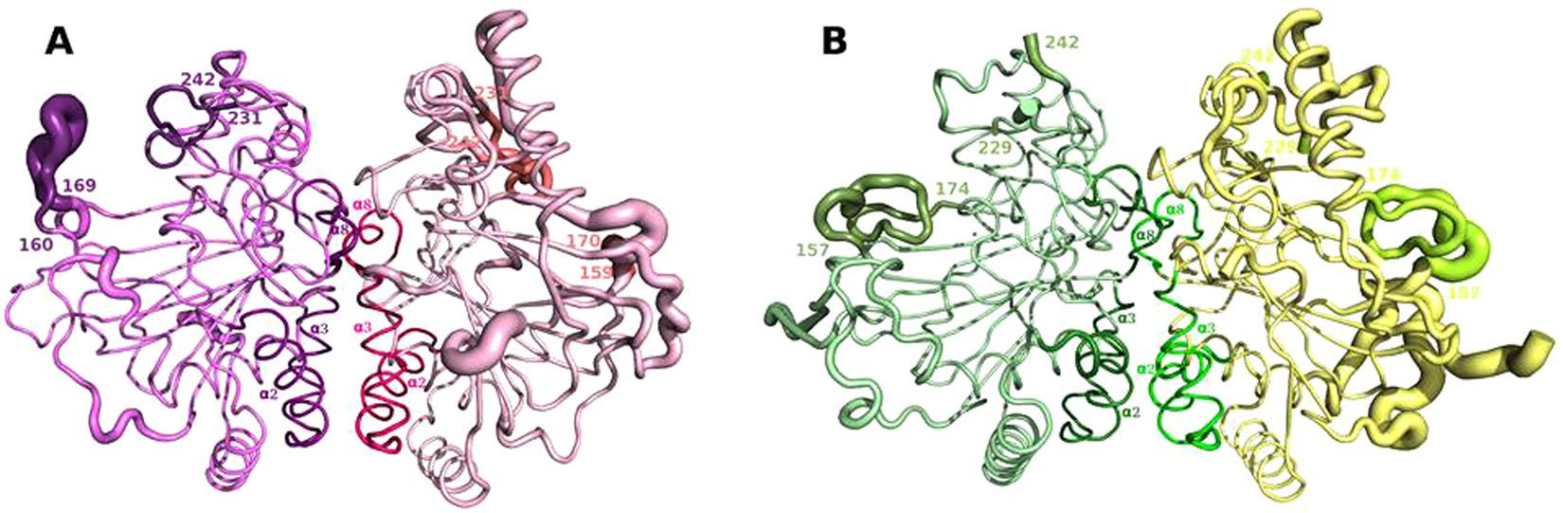
B-factor putty representation of the dimeric interface of KDO1 and KDO5. The thickness of a region is proportional to its local B-factor and thus its flexibility. (**A**) KDO1 dimer in shades of pink (monomers A and B of the KDO1-Fe-α-KG structure). (**B**) KDO5 dimer in shades of green (monomers A and B of the KDO5-Fe-α-KG structure). Helix α2, α3 and α8 at the dimeric interface, lid and adjacent loop are highlighted.

The conformation of the four monomers that form the asymmetric unit varies locally from monomer to monomer, probably influenced by lattice interactions, and varies on binding of substrates. Predictably, the dimer interface is well defined while the more peripheral portions are more agitated (Fig. 1).

The structures are characterized by several flexible loops (Table S3). The two loops (160–169 and 231–242) for KDO1 (Fig. 1A) and (157–174 and 232–241) for KDO5 (Fig. 1B), controlling the entrance to the catalytic site are among the most flexible and their flexibility is transmitted to the surrounding zone. In KDO5, the flexible loop 102–120 is also part of the entrance of the catalytic site. It is worth noting that such loops (±230–±240), already described on X-ray structures of αKAO, are referred to as lids, as they probably aim to shield the substrate from bulk solvent access to enable the reaction to proceed^17–19^. The other loop enclosing the active site (±160–±170) is named the adjacent loop in our study (Fig. S3).

### Comparison of KDO1 and KDO5 active sites

**Figure 2:**
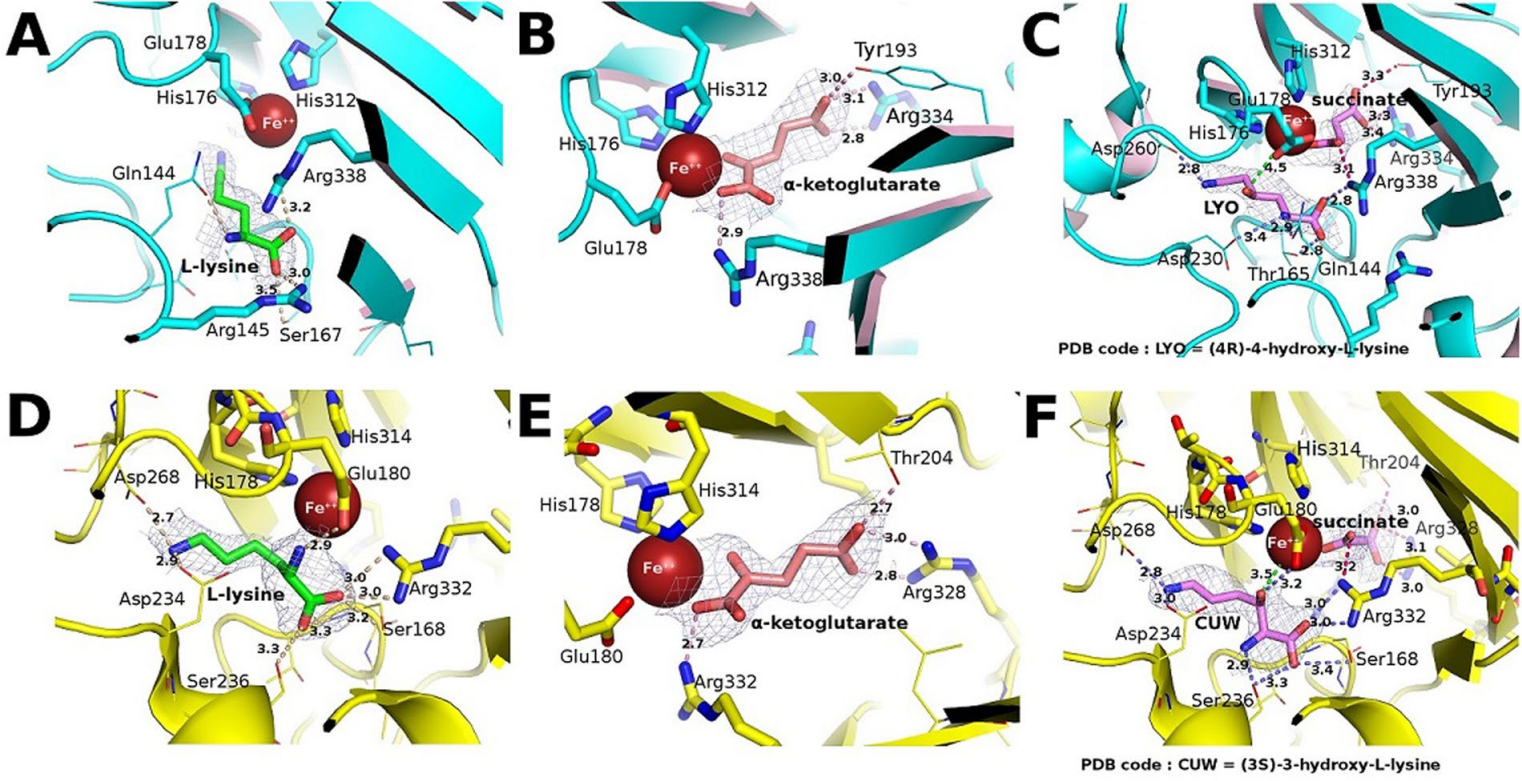
Comparison of the binding sites of KDO5 and KDO1, with the 2F_o_-F_c_ electron density (contoured at 0.5 σ) of the bound substrates and products. (**A**) KDO5 in complex with L-lysine (monomer C). (**B**) KDO5 in complex with α-KG (monomer A). (**C**) KDO5 in complex with (4*R*)-4-hydroxy-L-lysine and succinate (monomer D). (**D**) KDO1 in complex with L-lysine (monomer C). (**E**) KDO1 in complex with α-KG (monomer B). (**F**) KDO1 in complex with (3*S*)-3-hydroxy-L-lysine and succinate (monomer B). Among the four molecules in the asymmetric unit, only the binding site with the best positioning in the electron density for the ligands has been illustrated. Identifying codes assigned for the small ligand compounds are indicated on Panels B and E.

We have selected the best defined monomers and the structures with best bound ligands (Tables S4 and S5). As KDO1 ligands have better overall electron density, they are thus better stabilized than the ligands for KDO5. In all four molecules of each of the KDO5 structures, an iron ion is present in the active site chelated by two His (176 and 312) and one Glu (178). In KDO5-lysine crystal structure within the binding site of monomer C (Fig. 2A), the carboxylate of L-lysine substrate is stabilized by salt bridges with the Arg338 (3.2 Å) and 145 (3.0 Å) in the active site, and positioned by one H-bond with Ser167 (3.5 Å). In α-KG bound structure, α-KG is best defined for monomer A (Fig. 2B). The carboxylate moieties of the co-substrate form salt bridges with Arg338 and 334 (2.9 Å; 2.8 Å and 3.1 Å); the α-ketoacid moiety binds the iron as bidentate; the terminal carboxylate interacts by H-bond with Tyr193 (3.0 Å). In KDO5, the ligands in the product complex are less well defined, reflecting their ability to escape from the active site at the completion of the catalytic event. The better defined products are located within monomer D. The (4 *R*)-4-hydroxy-L-lysine is stabilized by salt bridges with Arg338 (2.8 Å), Asp230 (3.4 Å) and Asp260 (2.8 Å), and makes H-bonds with Gln144 (2.8 Å), Thr165 (2.8 Å). The C4-OH group is close to the iron (4.5 Å) (Fig. 2C). The succinate resultant from the oxidative decarboxylation of the α-KG, makes H-bond Tyr193 (3.3 Å), and establishes salt bridges with Arg338 (3.1 Å), Arg334 (3.3 and 3.4 Å) and with the iron (Fig. 2C).

The binding sites are different between KDO5 and KDO1. In KDO5 the pocket is characterized by a neck defined by four residues, namely, Trp112, Arg145, Asn232 and Asp230. Of these, Arg145 is instrumental in the recognition of L-lysine, as it is able to attract the substrate towards the end of the pocket where it can be maintained during the reaction (Fig. 2A).

While a ferrous ion is present in the KDO5 apo structure, it is not the case in KDO1 apo as the metal is lost during the purification from the KDO1 preparation used to grow the crystals. After crystal soaking with FeSO_4_ the two His residues (178 and 314) and Glu180 in the KDO1 active site recapture an iron. The entrance of the catalytic pocket of KDO1 is bifurcated, and the main opening does not match the one of KDO5. In KDO1, the L-lysine complex shows a well ordered lysine in monomer C **(**Fig. 2D). The interacting residues are Glu180, Arg332, Asp 234, Asp 268 (salt bridges 2.9 Å; 3.0 Å and 3.0 Å; 2.9 Å; 2.7 Å), Ser168 and Ser236 (H-bonds 3.2 Å and 3.3 Å; 3.3 Å). The Nε is restrained at the bottom of the pocket by two salt bridges with the Oδ2 of Asp268 and the Oδ1 of Asp234, while in KDO5 the Nε is stabilized *via* a water molecule in interaction with Asp260. The size of the KDO1 pocket is smaller than that of KDO5 due to Ser168 (instead of Gly). In the α-KG crystals, the structure shows a well ordered α-KG in monomer B. The α-KG makes salt bridges, with Arg332 (2.7 Å), Arg328 (2.8 Å and 3.0 Å) and H-bond with Thr204 (2.7 Å), while its α-ketoacid moiety binds the iron as bidentate (Fig. 2E). For KDO1, the best product is located within the active site of monomer B (Fig. 2F). The (3*S*)-3-hydroxy-L-lysine makes salt bridges with Glu180 (3.2 Å), Arg332 (3.0 Å and 3.0 Å), Asp234 (3.0 Å) and Asp268 (2.8 Å), and H-bonds with Ser168 (3.4 Å) and Ser236 (2.9 Å and 3.3 Å). The C3-OH is located close to the iron (3.5 Å). The succinate makes H-bond with Thr204 (3.0 Å), and salt bridges with Arg332 (3.2 Å), Arg328 (3.0 and 3.1 Å) and the iron (Fig. 2F).

In comparing the product complexes of KDO1 and KDO5, we found that the electron densities of the ligands are generally better for KDO1, except for monomer D of KDO5. The hydroxylysines overlap is slightly off by 3 Å, as would be expected given that different positions are hydroxylated (Fig. S4). In KDO1, the Nε of hydroxylysine is better stabilized than in KDO5, because the two H-bonds (Asp234 and Asp268) (Fig. 2E) stabilize the substrate after interacting with a water molecule (H_2_O-562: Table S5). In the case of KDO5, the Nα of the product does not maintain the interaction with the equivalent water molecule as the peptide amide becomes positioned where the water molecule was located, deeper in the pocket. The position of the active site residues in both KDO1 and KDO5 remains unchanged between the substrate and product complexes.

### Crystallographic considerations

Although the resolution of the KDO5 and KDO1 structures in complex with the lysine substrate are comparatively high, the electron density for the substrates is not well defined. For both KDO1 and KDO5, in the presence of α-KG and lysine, the reaction takes place and it was therefore impossible to obtain crystals with both substrates simultaneously. However, we could obtain crystals in the presence of the two products resulting from the reaction. In the absence of the α-KG, unlike what is observed for the lysine-KDO1 complexes, the lysine is positioned differently in the four molecules of lysine-KDO5, suggesting that this substrate is not properly stabilized. The flexibility of the N-terminus of KDO5 might have a role in the access to the active site. The first residue that can be positioned in any KDO5 structure is Ala13 in monomer C of the apo form; for KDO1 the first ordered residue is Glu30 in monomers A and D of the apo form.

### Relationship between members of the Clavaminate synthase-like family

According to our knowledge, αKAOs encompass 11 phylogenetically unrelated enzyme families according to the InterPro database^20^ (Table S6). Enzymes that transform free L-amino acids, such as KDO1 and KDO5, are found in four different families represented by enzymes called VioC, MpDO, SadA and GriE (Table S6)^17,21–24^. Because they possess different folds (Table S6), alignment of their whole structures is not possible. However, a structural alignment of the 2-His-1-carboxylate facial triad of their structures is possible and can help to define a spatial arrangement of functional groups (Fig. S5).

**Figure 3:**
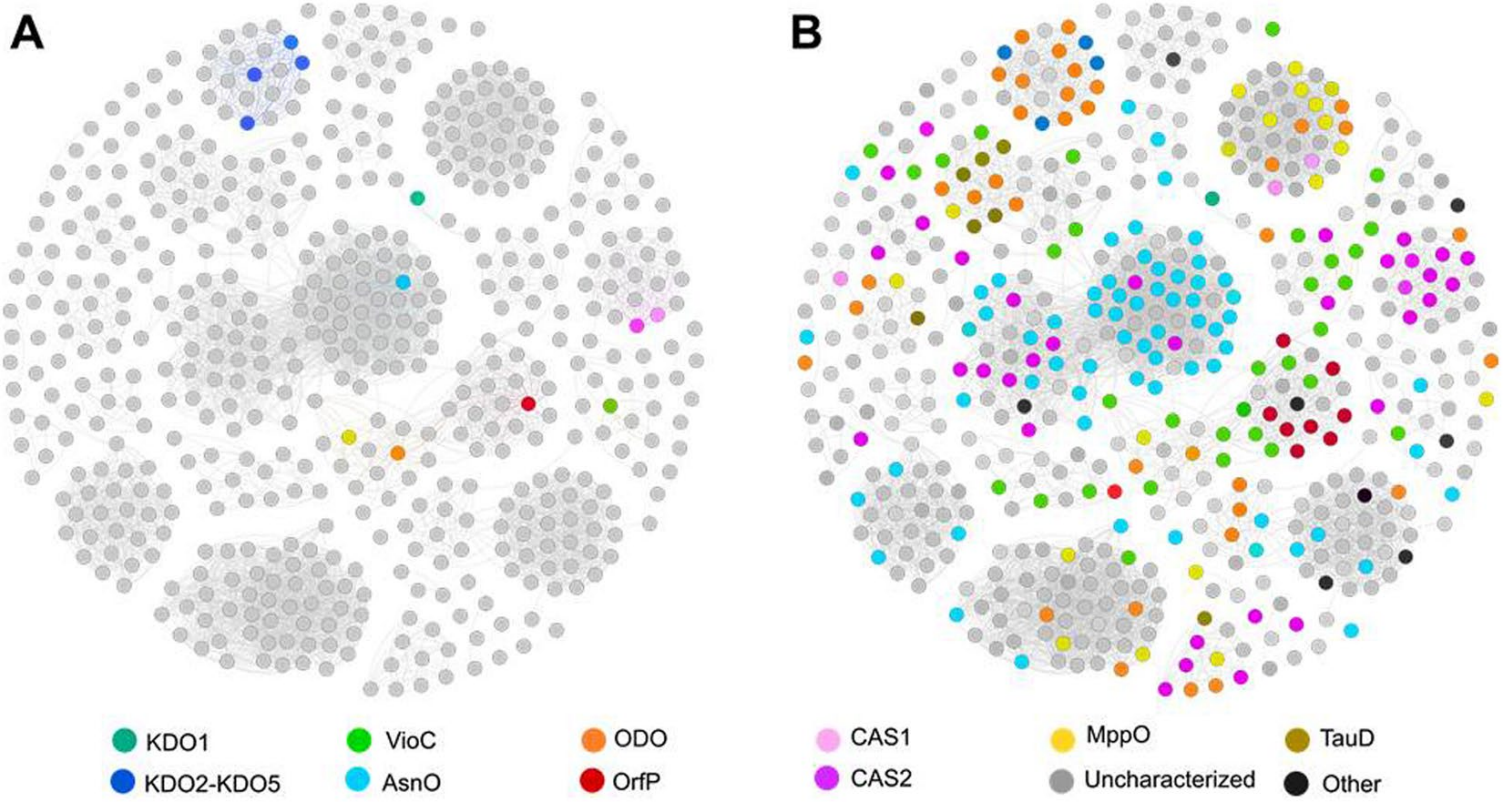
Sequence Similarity Network (SSN) of the Clavaminate synthase-like family (IPR014503 InterPro family) from the αKAOs superfamily. Nodes represent proteins and edges are the links between those proteins that have at least 50% sequence identity. (**A**) Experimental data retrieved from Swissprot mapped on the SSN. KDO5 and other KDO2-4, characterized previously^16^ are colored in dark blue. (**B**) Annotations from Uniprot mapped on the SSN. The network is visualized with Gephi (Bastian, “Gephi: An Open Source Software for Exploring and Manipulating Networks”, AAAI Publications, Third International AAAI Conference on Weblogs and Social Media, 2009).

KDO1 and KDO5 belong to the Clavaminate synthase-like family (CSL), which is classified in the IPR014503 InterPro family. In order to analyze the sequence diversity and the relationships between proteins of this family, we performed a sequence similarity network (SSN) using the EFI-EST^25^. In SSNs, the most related proteins are grouped together in clusters. The network reveals five highly populated sub-families for which there was no reported experimental data (Fig. 3A). Mapping the annotation retrieved from Uniprot shows mis-annotation for the family as close homologs were assigned different functions or remote homologs are assigned identical functions (Fig. 3B). For instance, the sub-family highly connected to the sub-family containing AsnO, brings together CAS annotated proteins. KDO5’s subfamily is fully disconnected from other CSL dioxygenases, showing an early evolution away from them. However, sequences in the KDO5 sub-family share on average 70% identity and are found in two different taxonomic classes (data not shown), indicating that, although these sequences have evolved, the proteins maintain the same enzymatic activity^16,26^.

### Active site profiles within CSL family

Enzymes catalyzing the same chemical transformation while sharing low sequence identity, present identical residues in their active site as evolutionary pressure tends to conserve the residues playing a critical role in the catalysis^27^. Because loop or subdomain insertions can disrupt sequence alignment, these conserved residues are better identified in protein structures. Previously, it has been shown that, in general, substrate specificity is linked to the presence of a few distinctive amino acids in the active site^28^.

**Figure 4:**
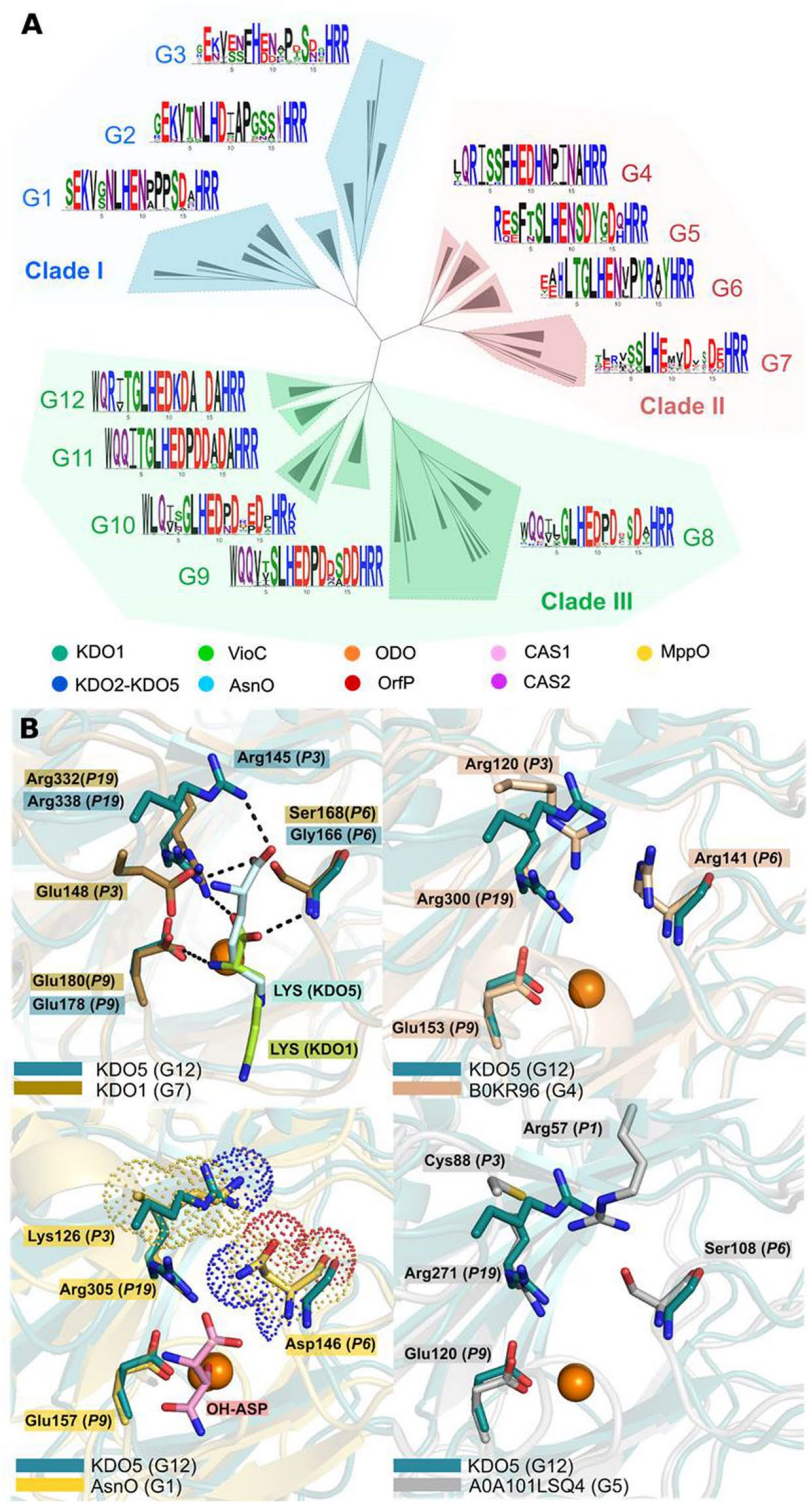
Dividing the CSL family into groups with similar active sites. (**A**) ASMC hierarchical tree containing 523 proteins, grouped in 3 clades (I, II, III). For each ASMC group (from G1 to G12), a logo sequence is depicted and represents the conservation of residues in the active site pocket. Proteins with known activities are indicated by a circle in the tree. (**B**) Regioselectivity among the CSL proteins. Superimposition of crystallographic structure of KDO5, chain C, in complex with lysine, with crystallographic structure of KDO1, chain C, in complex with lysine (top left), with a representant of group G4 (model with Uniprot ID B0KR96) (top right), with crystal structure of AsnO in complex with hydroxy-aspartate (PDB code 2OG7) (down left) and with a representant of G5 (model with Uniprot ID A0A101LSQ4) (down right). Residues interacting with the carboxylate and amino group of the substrates are indicated in stick, along with their position in the logo. Black dashed lines indicate interactions between substrates and proteins.

To understand what makes CSL dioxygenases specific to a range of substrates, we carried out an analysis of the active sites of all its members. Homology modeling and subsequent classification of active sites was performed using ASMC (Active Site Modeling and Clustering) software^29^. The resulted “Active site tree” was divided in 12 groups (referred to as G1 to G12), each containing between 15 and 91 sequences. The three-dimensional superposition of modeled and crystallographic-resolved active sites within each group was projected linearly to form conservation patterns called logos (Figs 4A and S6). Thus, 3D-motifs found in the active sites can be easily read on the logos. All logos have the conserved triade His, Glu/Asp, His (respectively in position *P8*, *P9* and *P17* of the logo) for iron binding. Arg in *P18* is highly conserved and forms a salt bridge with the carboxylate group of α-KG/succinate. Two other residues are very conserved: Leu in *P7* and Arg in *P19*, which delimit two distinct pockets that host α-KG/succinate and the substrate/product (Fig. S7). Arg in *P19*, conserved in all ASMC groups, interacts with the carboxylate group of the substrates. A large majority of the groups have a glutamine or a glutamate in *P2*. These residues (Gln in KDO5, VioC, OrfP, ODO, or Glu for AsnO, and KDO1) interact with the α-NH_2_ of the amino acid substrates. Only a few exceptions are noted in the family, like CAS 1 from G7 (Leu in *P2*), where proclavaminate substrate is found deeply in the pocket, or proteins from G10.

### C4 regio-selectivity in KDO5 and other CSL dioxygenases

The logo of KDO5’s group (*i*.*e*. G12) is highly conserved and shows an arginine in position *P3* which corresponds to Arg145 (Fig. 4A). As observed in crystallographic snapshots, it interacts through a salt bridge with the carboxylate group of the L-lysine substrate and might be responsible for the regioselectivity of the reaction (Fig. 4B). Indeed, this residue, absent in KDO1 (C3-regioselective) (Fig. 4B), seems to be a key residue for positioning the substrate deeply in the pocket and bringing the C4 close to the iron, resulting in a C4-regioselectivity. Force constants have been calculated on crystallographic structures of different members of the CSL family (VioC, AsnO, CAS, OrfP, KDO1 and KDO5)^17,19,30–32^. Our analysis revealed that Arg145, even when not situated on a rigid secondary element, is part of this very rigid core, underlying its high importance for KDO5 activity (Fig. S8).

Arg in *P3* of the logo is also found in CAS1 (G7) crystallographic structure^18^. The side chain of this residue (Arg115) points toward the carboxylate of the substrate, and might be involved in binding with the substrate *via* a water molecule^31^. As G12 group, CSL dioxygenases from G4 also present an Arg in *P3* of the logo (Fig. 4B), suggesting that these enzymes might also catalyze the C4-hydroxylation of an amino acid. Arg in *P3* of G12 and G4 is part of an electropositive patch that includes the Lys in *P11* (G12) and His in *P11* (G4) (Fig. 4B).

Interestingly, groups from Clade I (*i*.*e*. G1, G2, and G3) show a Lys in *P3*. This Lys does not influence the electrostatic properties of the pocket region that hosts the carboxylate group of the substrate, because this residue is buried by Asn in *P6*, a residue conserved in this clade (Fig. 4B). Consequently, the type of residue in *P3* for proteins of Clade I should have no direct influence on the regioselectivity of the reaction.

For G5 proteins, the three-dimensional motif formed by Arg in *P1* and the catalytic residues (His in *P8*, His in *P8*, Glu/Asp in *P17*) is the same as the one found in the proteins of G4 and G12, where an Arg is found in *P3*. Even if these two Arg (in *P1* of G5 and *P3* of G4 and G12) are not found at the same position in sequence, their side chains point in the same direction in 3D (Fig. 4B). Thus, because enzymes from G4 and G12 groups share this common 3D-motif with G5, it is expected that they will have the same regioselectivity towards the substrate.

Taxonomic analysis of ASMC groups shows that enzymes predicted to hydroxylate in C4 (*i*.*e*. G4, G5 and G12 groups), are found in restricted taxonomic branches (Fig. S9) and might have appeared relatively recently (*i*.*e*. Proteobacteria). By contrast, the oldest taxonomic levels, such as Actinobacteria phylum, present a great diversity of CSL dioxygenases.

### A sub-pocket for selecting the type of substrate

**Figure 5:**
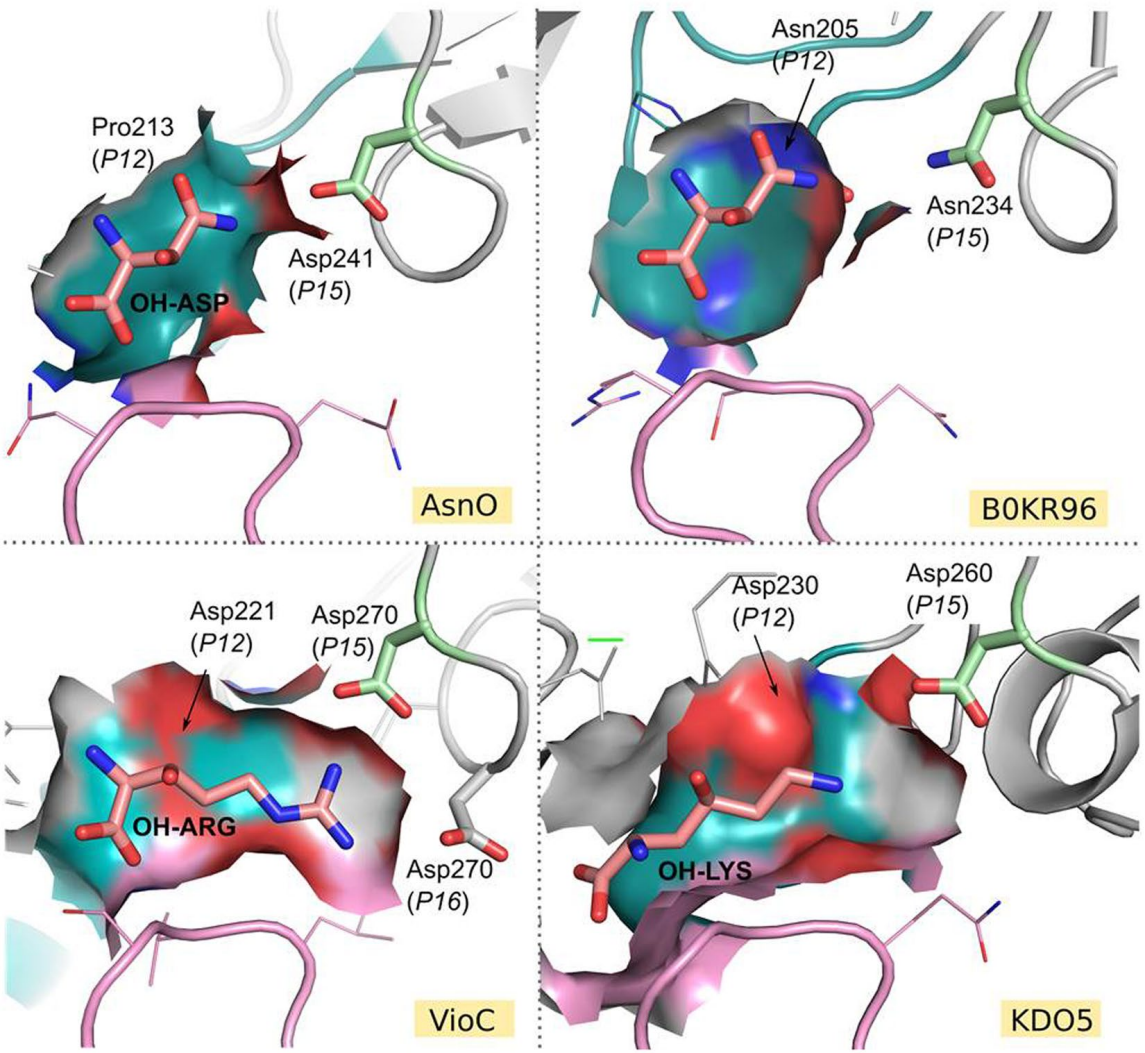
Depiction of the sub-pockets in the CSL family. For Clade I, represented by AsnO, the sub-pocket is formed by 3D motif PPSD in *P12* to *P15* of the active site logo. For Clade II, a representative of the G4 group, a model of protein with Uniprot ID B0KR96, was chosen. The sub-pocket is composed of residues NPIN. To get an idea of the size of substrate that can host the protein, Asn from AsnO was positioned at the center of the binding pocket. For Clade III, represented by VioC and KDO5, the sub-pocket is formed by 3D motifs DSDD and DA-D respectively. Residue in *P15* of the logo is represented in green. The flexible lid is colored deep blue teal with atoms colored by elements. The flexible lid occupies a large part of the binding pocket, which is represented in surface. The adjacent loop is colored in pink.

A conserved 3D-motif DXXD in *P12* to *P15* of the logo is found in all groups of Clade III and in two groups of Clade II (G5 and G7). Asp in *P12* and Asp in *P15*, conserved respectively in 55% and 76% in CSL proteins are spatially close, the latter being also very conserved in these same ASMC groups (and in G1). These two Asp form a negatively charged sub-pocket into which the side-chain of the substrate fits, as observed in the crystallographic structures of KDO1, KDO5, VioC, OrfP and CAS (Fig. 5). DXXD is also observed in models of ODO and MppO, both active towards substrates with electropositive side chains. More broadly, it is expected that this motif/sub-pocket is present in CSL dioxygenases active towards substrates with positively charged side chains. In Clade III, a highly conserved Asp in *P10* takes part of the electronegative potential of the sub-pocket and interacts through electrostatic interactions with the residues in *P3* (*i*.*e*. Arg in G12 or Gln/Glu in the other groups of Clade III).

Interestingly, G4 and G6 groups do not display this negatively charged sub-pocket, but a hydrophobic (motif NPIN from *P12* to *P15*) and positively charged (motif PYRA/V) sub-pocket respectively (Fig. 5). The consequent assumption is that enzymes from G4 might catalyze the hydroxylation of hydrophobic substrates, while those from G6 might transform amino acids with a negatively charged side chain, such as glutamate or aspartate. The sub-pocket we describe here cannot be found in the crystallographic structures of other αKAO known to transform hydrophobic substrates or aspartate, such as P3H2, P4H, AspH, all classified in another InterPro family (IPR007803) (Table S6). Indeed, the substrate/product binding pocket of these enzymes are not structured the same way as in CSL enzymes (Fig. S10).

In summary, the sub-pocket disclosed in our models, formed by the residue *P12* of the flexible loop and *P15*, is critical for the selection of the type of substrate. In VioC structure, this sub-pocket, negatively charged, is strengthened by the presence of an additional Asp in position *P16*, very conserved in the G10 group, interacting with the bidentate guanidinium of the arginine substrate side chain (Fig. 5). In G6, a very conserved Tyr is found in *P16*, suggesting that this residue might play an important role, like Asp in *P16* of VioC, in interacting with a large substrate (sterically equivalent to an arginine). In groups of Clade I, DXXD motif from *P12* to *P15* is absent. However, an Asp is found in *P15* of G1, and partially in G3, which might play a role in substrate positioning, as exemplified by the short distance (3.6 Å) observed in AsnO structure in complex with (2*S*,3*S*) hydroxy-asparagine.

### Induced lid motion upon substrate binding

Previous studies have highlighted that the flexible lid can change its conformation upon substrate binding^19^, be partly disordered when complexed with ligands^18^, or completely disordered in the apo-form, but become ordered when the substrate is bound^17^. It was hypothesized that the lid shields the substrate from bulk solvent access to enable the reaction and is involved in the substrate selection^17–19^. The diversity in terms of sequences, shape and length of this flexible lid among the family could be another element to explain the high substrate specificity of CSL dioxygenases. Models belonging to the G1 group show the same lid length as AsnO, whereas models from the G2 group have a loop without a well-defined structure which is the same length as the KDO5 lid. Thus, despite a large variability in terms of length and sequence (Table S7), a conserved region is involved in the sub-pocket formation after substrate binding.

Unfortunately, the lack of resolution for the lid region of the KDO5 crystallographic structures makes it difficult to interpret the exact roles of residues other than *P12*. In VioC, a Ser (Ser224, *P14*) establishes a H-bond with a Gln (Gln137, *P2*) which, in turn, positions the latter in the active site by interacting with the Nα of L-arginine substrate. This induces the stereoselectivity of the reaction. In the monomer C bound to lysine (Fig. S11), the flexible loop is better defined until residue Tyr233 (not in logo). On it, Asn232 (a residue which should appear in *P14* of the logo, but is not shown on it as it could not be superimposed correctly with the reference pocket) (Fig. S11) is orientated in a certain way, so that its interaction with the Nα group of L-lysine substrate positions the latter to perform the observed stereoselectivity. Thus, lid closure could be directed by a H-bond between *P14* and the Nα of the amino acid substrate, either directly, as exemplified by KDO5, or indirectly, as observed for VioC.

### Influence of the adjacent loop

Neighboring loops to the flexible lid might also be involved in substrate selection. For KDO1, the lid is well defined both in the apo and complexed forms, but the adjacent loop, from Ala160 to Ser171 (not in the logo except for *P5* and *P6*, respectively residues 167 and 168) undergoes large conformational changes. Among all CSL dioxygenases whose crystal structures have been reported so far, this is the first time such a loop motion is observed. Its motion seems to be induced by α-KG binding as this loop is closed in apo and complexed forms with products and lysine, but opened when α-KG is bound (Fig. S12). In the open form, lysine or α-KG can easily access the active site and the iron. Induced fit motion between the adjacent loop and the flexible lid seems to be correlated with polar interactions between His164 (not in logo) and Glu235 (*P13*), both of which are distant from 4.1 Å (Fig. S12A). In the closed form, the lysine or hydroxy-lysine/succinate are covered by the adjacent loop and the active site is locked by interactions between Thr167 (*P5*) and Ser236 (*P14*) (Fig. S12B). In KDO5’s monomer C in complex with lysine, the active site is locked by interaction between Thr165 (*P5*) and Asn232 (supposed to be on *P14*, see above paragraph). *P5* is a conserved position in the CSL family where Thr, Ser or Leu are frequently found (34%, 25%, 13% respectively). On the contrary, *P13* and *P14* are not conserved in the logo probably because of the large variety of length, shape and sequence compositions of the lid. For KDO1, the adjacent loop has a direct impact on positioning the substrate in the active site as Ser168 (*P6* in the logo) makes a H-bond with the carboxylate group of the lysine. In KDO5, Gly166 (*P6*) and Ser167 (*P7*) also stabilize the carboxylate group of the substrate, like Asn146 (*P6*) in AsnO^19^.

### Conservation in the dimeric interface

Dimers have been described previously for other αKAOs^33^. The similarity of the dimeric interface between KDO1 and KDO5 (Fig. 1) suggests that for the proper functioning of these enzymes, an interface may be needed. Such an assembly is also observable for the crystallographic structures of VioC, OrfP and AsnO, in which the same dimeric arrangement is mediated by two helices. A strong dimeric interface may provide a stabilizing force to allow flexible elements, such as the loops surrounding the active site, to adapt to structural requirements for a fast and stereoselective catalysis^34^.

Throughout the family, the residues that are part of the interface are found to be very diverse (Fig. S13A). However, an Asp, positioned at the middle of the interface for VioC, AsnO, KDO1, KDO5 and OrfP crystal structures, is conserved in 70% of the proteins (Fig. S13B). In KDO5, this Asp (Asp187) is involved in a H-bond network connecting residues from one chain with two residues from the other chain (Fig. S13C). This network creates a small cavity in which a hydrophobic residue (Ile in KDO5) from the other chain is hosted (Fig. S13D).

## Conclusion

The CSL family brings together Fe(II)/αKAOs, some of which are known to catalyze the hydroxylation of amino acids and derivatives with a high specificity. We have determined the structures of two regiodivergent lysine αKAOs in various states. These results allowed us to enrich the structural and computational analysis in the whole family to try to decipher the key determinants of selectivity. Our analysis strongly suggests that the substrate specificity together with the high stereoselectivity observed in this family are related to two structural features: a flexible loop (lid) and a sub-pocket. Our work pinpointed a particular residue that correlates with the regioselectivity. Although the sequence and length of the lid vary greatly within the family, our crystallographic structures confirm its dynamic nature and suggest a common behavior of closing upon substrate binding to form a sub-pocket surrounding the substrate, and re-opening to release the products. Our results also highlight the flexibility of an adjacent loop, enclosing the active site. A constant feature observed in this family is a conserved dimeric interface, with the flexible lid positioned at its opposite end. We therefore suggest that this stable interface is a key feature necessary to allow the lid flexibility and therefore a fast, regio- and stereoselective reaction. In summary, our analysis provides new insights into the specificity of the CSL enzymes and can guide further functional exploration for the discovery of αKAOs with new activities.

## Methods

### Protein preparation

The cloning, enzyme production and purification have been described elsewhere^16^. Oligonucleotides were purchased from Sigma-Genosys (St. Quentin Fallavier, France) and specific extensions added for cloning into pET-22b(+) (Novagen) including a hexa-histidine tail. The plasmid was introduced into E. coli BL21(DE3) for over-expression. The KDOs were purified on a Ni-NTA affinity column as reported previously^35^. Protein concentration was estimated using the Bradford method, and the purity was evaluated by SDS-PAGE electrophoresis. Aliquots of the purified protein were stored at −80 °C.

### Protein oligomeric state determination

Analytic gel filtrations were performed on a Superdex 200 Increase 10/300 GL column by injecting 100 µL of the purified protein with the following mobile phase: 50 mM Tris-HCl pH 8.0, 50 mM NaCl, 10% glycerol at a flow rate of 0.3 ml/min. The oligomerization state of the targeted protein was determined according to its elution volume compared to a calibration curve obtained with proteins of known global molecular weight.

### Crystallization, data collection and structure determination of KDO1 and KDO5

The initial screening was carried out on a mosquito crystal liquid handler (TTP Labtech, Cambridge, UK) using sitting drops of 150 µl of protein. For KDO1, larger crystals were grown using equal volume drops of KDO1 at 9.3 mg/ml in 0.050 M Tris-HCl, 0.2 M NaCl, 0.001 M DTT, pH 8.0 and of reservoir solution, by hanging drop vapor diffusion. For KDO5, crystallization experiments were carried out using equal volume drops of KDO5 at 5 mg/ml in buffer, 0.050 M Tris-HCl, 0.2 M NaCl, 0.001 M DTT, pH 8.0 and of reservoir solution, by sitting drop vapor diffusion. Crystals were improved by the clever technique of being transferred to a cryosolution (Tables S1 and S2), and then flash cooled in liquid nitrogen. To obtain the KDO1 complexes, the crystals were soaked in either a solution of FeSO_4_/dithionite and L-lysine or FeSO_4_/dithionite and α-ketoglutarate or FeSO_4_/dithionite, Na succinate and (3 *S*)-3-hydroxy-L-lysine. The ligand soaking was carried out directly in the cryosolution. To obtain the KDO5 complexes, the crystals were soaked in either a solution of FeSO_4_ /dithionite and L-lysine, or FeSO_4_/dithionite and α-ketoglutarate, or both simultaneously. The concentration of the ligands and the soak time were optimized to obtain a good occupancy of the ligands with minimal damage to the crystals (Tables S1 and S2).

X-ray diffraction data sets were collected at the SOLEIL Synchrotron (Saint Aubin, France), on beamline Proxima 1 of a Dectris Pilatus 6 M detector, or on beamline Proxima 2 A of a Dectris EigerX 9 M or ADSC Q315r detector (Tables S1 and S2).

Data processing was carried out at the synchrotron facility using XDS with the xdsme script^36^. To solve the KDO1 structure, molecular replacement was carried out using PDB entry 2WBO^17,37^ as the model. The electron density maps were viewed and fitted in COOT^17,37^ and fitted with the sequence corresponding to UNIPROT C7QJ42. To solve the KDO5 structure, since molecular replacement with various models failed, a heavy-atom soaking approach was followed. The crystals used to solve the structure were obtained by being soaked for 20 mins. in 26% polyethylene glycol 3350, 0.2 M Imidazole malate, 0.15 M Li_2_SO_4_ at pH 7.0 with 0.2 mM K_2_ReCl_6_ in a cryosolution composed of 30% CM5. A data set spanning 360° of data was recorded at the rhenium L3 absorption edge. Four rhenium sites were located using SHELXD^38^. Initial Re-SAD phases were estimated and refined using PHASER^39,40^ and then improved and extended by density modification, including NCS averaging, using the PARROT program^39,40^. A first model could be built automatically by BUCCANEER^37,41^ and the rebuilding completed using COOT^17,37^ with the sequence corresponding to UNIPROT J3BZS6.

The structures were subjected to various cycles of rebuilding COOT and refinement with BUSTER^42^. To take into account the anisotropic fall-off in resolution for KDO5-Lys and KDO5-α-KG datasets, STARANISO^43^ was used. These data were used in all subsequent model building and refinement cycles.

The structures have been analyzed with PISA and PLIP^44^ servers to calculate the surface buried in the interfaces and the interactions made by the ligands^45^. Of the four monomers in the asymmetric unit, the monomer with the lowest Local Ligand Density Fit (LLDF) score and the strongest electron density for the ligand is considered to be the most representative^46^.

### Sequence similarity network

The initial set of proteins, containing all members of the Clavaminate synthase-like InterPro domain (IPR014503), was collected in May 2017 (587 sequences). Homologs to KDO1 and KDO5 retrieved from Uniprot using a Z-score threshold value of 150 were added to the set. The list of proteins (UniProt IDs) was provided to SSN-EFI (http://efi.igb.illinois.edu/efi-est/)^25^. Sequences and annotation information from the UniProt database were retrieved and mapped on the SSN.

### Active site classification

To the set of CSL family, we added 153 homologs to KDO1 and KDO5 retrieved from Uniprot using a threshold Z-score value of 150. The entire set was clustered based on 100% sequence identity to obtain a non-redundant set of 608 proteins. Only proteins sharing at least 22% of sequence identity with at least one alpha αKAO for which structures are available were kept (see following paragraph). In total 523 proteins were used for the active site classification, using ASMC software with three main steps^29^. Firstly, homology models for our set were built using structures templates, the crystal structures of CAS1 in complex with α-KG and proclavaminic acid (PDB 1DRT, chain A), VioC with (2*S*, 3*S*)-hydroxy-arginine (2WBQ, A), AsnO with succinate and (2*S*, 3*S*)-hydroxy-asparagine (2OG7, A), OrfP with succinate and (3*R*, 4*R*)-dihydroxy-L-arginine (4M2G, A), KDO1 and KDO5^17,19,31,32^. Structures of VioC with L-arginine and α-KG (6ALM) and NapI (6DAW) were not yet released in the PDB^9,11^ at the time of our homology modeling work. Secondly, detection of pockets was performed on KDO5. Above the 12 predicted pockets, we selected the pocket that contained the catalytic (His, His, Glu/Asp) and the residues binding L-lysine. This pocket contains 28 residues and corresponds to the pocket surrounding the substrate. Among them, we kept residues whose Cα atom is within a distance of 12.5 Å from the catalytic glutamate. In total, the reference pocket is composed of 19 residues. The 19 equivalent residues were selected on VioC crystal structure to form the active site reference. VioC was chosen as the reference structure as it is average sequence according to the SSN. Finally, the 523 sequences were structurally aligned on VioC structure. Residues aligned with the 19 residues the active site reference were extracted to form a multiple sequence alignment of 523 sequences which were subsequently classified.

### Comparing lids and dimer interfaces between members of the family

Homology models built were structurally aligned on KDO5 structure. We examined the residues that are structurally aligned with the extremities of the lid loop highlighted in this work. The lid corresponds to residues from 230 to 242 in KDO5.

Dimer interfaces formed by αKAOs monomers for each homology model were examined using an alternative version of the ASMC option (unpublished). In this version, the residues in contact between chain A and B of the KDO5 crystal structure are provided to ASMC to form logo sequences. Interface residues are retrieved using the BioPython/GetInterfaces.py script (provided by www.blopig.com). Residues are considered in contact if any of their atoms are less than 4.5 A from any atoms of residues of the other chain. The interface neighbor cutoff was defined as 10 Å.

## Electronic supplementary material

Supplementary information
