# Supplementary material for: Structural Studies based on two Lysine Dioxygenases with Distinct Regioselectivity Brings Insights Into Enzyme Specificity within the Clavaminate Synthase-Like Family
Source: Sci Rep. 2018 Nov 8;8:16587. doi: 10.1038/s41598-018-34795-9 (PMC6224419; doi:10.1038/s41598-018-34795-9)
Supplement: Supplementary file 1 — Supplementary information [file 41598_2018_34795_MOESM1_ESM.pdf]

Supplementary information

Structural Studies based on two Lysine Dioxygenases with Distinct Regioselectivity Brings Insights Into Enzyme Specificity within the Clavaminate Synthase-Like Family

Karine Bastard,[1⊥] Tatiana Isabet,[2⊥] Enrico A. Stura,[3] Pierre Legrand[2] and Anne Zaparucha[1]*

[1]: Génomique Métabolique, Genoscope, Institut François Jacob, CEA, CNRS, Univ Evry, Université Paris-Saclay, 91057 Evry, France
[2]: Synchrotron SOLEIL, L'Orme des Merisiers, Saint-Aubin, BP 48, 91192 Gif-sur-Yvette, France
[3]: CEA, Institut des Sciences du Vivant Frédéric Joliot, Service d'Ingénierie Moléculaire des Protéines (SIMOPRO), Université Paris-Saclay, Gif-sur-Yvette 91190, France
[⊥]: these authors contributed equally.
Correspondence should be addressed to Anne Zaparucha (anne.zaparucha@genoscope.cns.fr)

Supplementary tables

Table S1 p. 2
Table S2 p. 5
Table S3 p. 9
Table S4 p. 12
Table S5 p. 15
Table S6 p. 18
Table S7 p. 22

Supplementary figures

Figure S1 p. 24
Figure S2 p. 25
Figure S3 p. 26
Figure S4 p. 27
Figure S5 p. 28
Figure S6 p. 30
Figure S7 p. 31
Figure S8 p. 32
Figure S9 p. 34
Figure S10 p. 35
Figure S11 p. 37
Figure S12 p. 38
Figure S13 p. 39

Supplementary tables

Table S1. Crystallization conditions and data collection statistics for KDO1

Structure	KDO1-APO[a]	KDO1-Fe-α-KG	KDO1-Fe-Lys	KDO1-Fe-products
**PDB code**	**6F2E**	**6F2B**	**6F2A**	**6F6J**
Crystallization	18% PEG 3350,	18% PEG 3350,	18% PEG 3350,	18% PEG 3350,
	.15 M Tris	.15 M Tris	.15 M Tris	.15 M Tris
	.3 M Na Acetate	.3 M Na Acetate	.3M Na Acetate	.3 M Na Acetate
	pH 7.5	pH 7.5	pH 7.5	pH 7.5
Cryoprotectant		20% glycerol	20% glycerol	20% glycerol
		20% PEG 3350,	20% PEG 3350,	20% PEG 3350,
	20% glycerol,	.2 M NaCl	.2 M NaCl	.2 M NaCl,
	20% PEG 3350,	.02 M Na Succinate,	.02 M Na Succinate,	.02 M Na Succinate,
	.2 M NaCl	.001 M $FeSO_4$,	.001 M $FeSO_4$,	.001 M $FeSO_4$,
	.15 M Tris, pH 7.5	.0001 M dithionite,	.0001 M dithionite,	.0001 M dithionite,
	20 sec. dip.	.05 M a-KG,	.05M L-lysine,	.02 M (3S)-3-hydroxy-L-lysine,
		.15 M Tris, pH 7.5,	.15 M Tris, pH 7.5,	.15 M Tris, pH 7.5,
		10 min. soak.	10 min. soak.	10 min. soak.
Data Collection				
Source	SOLEIL	SOLEIL	SOLEIL	SOLEIL

Beamline	Proxima-1	Proxima-1	Proxima-1	Proxima-1
Wavelength	0.9786	0.9786	0.9786	0.9786
Space group	*P1*	*P1*	*P1*	*P1*
Unit-cell (Å, °)	56.7 68.1 110.2	56.8 68.0 110.4	56.9 67.0 110.8	56.95 67.4 110.7
	107.8 102.8 93.2	107.8 102.8 93.5	107.5 102.8 93.7	107.6 102.9 93.6
Z'	4	4	4	4
Resolution (Å)	1.9(hires)	2.0(hires)	2.0(hires)	2.0(hires)
$CC_{1/2}$[b] (%)	99.7(66.8)	99.8(55.4)	99.5(57.2)	99.8(73.8)
$<I/\sigma(I)>$	10.2(1.31)	10.9(1.22)	8.3(1.19)	13.6(1.38)
R_{meas}[c] (%)	11.7(102.4)	10.0(112)	11.8(124)	8.3(83.3)
R_{factor}[c] (%)	10.4(86.4)	8.9(94.7)	10.3(102)	7.5(68.5)
Complete (%)	98.5(95.6)	98.8(93.7)	97.9(92.0)	99.0(93.1)
Multiplicity	4.65(3.45)	5.0(3.4)	4.02(2.96)	5.85(3.0)
Refinement	BUSTER[d]	BUSTER[d]	BUSTER[d]	BUSTER[d]
Resolution (Å)	38(1.95)-1.9	40(2.05)-2.0	20.2(2.05)-2.0	40(2.05)-2.0
No. of reflections	117663(8021)	101786(6981)	100086(6908)	101618(6945)
R_{work} (%)	17.7(22.9)	17.1(23.1)	20.3(22.3)	19.4(21.8)
R_{free} (%)	20.3(24.7)	20.1(24.5)	23.1(24.3)	22.5(24.7)
RMSD bonds				

Lengths (Å)	0.010	0.010	0.010	0.010
Angles (°)	0.97	1.00	1.04	1.02
Ramachandran				
favored	97.0 %	98 %	99 %	99 %
outliers	0	0	5	6
Interfaces[e]				
A-B (Å^2) / CSS	1375.6/ 0.918	1387.5 / 0.297	1385.0 / 0.392	1377.5 / 0.466
A-C (Å^2) / CSS	330.0 / 0.000	292.4 / 0.016	293.3 / 0.000	311.9 / 0.000
A-D (Å^2) / CSS	323.8 / 0.000	462.3 / 0.016	312.7 / 0.000	332.9 / 0.000
C-D (Å^2) / CSS	1386.1 / 0.918	1387.8 / 0.297	1406.4 / 0.392	1395.8 / 0.466

[a] The structure was solved by molecular replacement using MOLREP with 2WBO[1] chain A as in the model. [b] $CC_{1/2}$: Data quality correlation coefficient[2]. [b] Data collection statistics are from XDS[3]. [d] BUSTER is a program from Global Phasing[4]. [e] Interface analyzed by PISA[5]. The area buried in the interface and the CSS, (Complexation Significance Score) indicates the significance of the interface for the assembly formation, with a maximal value of 1, are reported.

Table S2. Crystallization conditions and data collection statistics for KDO5

Structure	KDO5-APO	KDO5-Fe-α-KG	KDO5-Fe-Lys	KDO5-Fe-products	KDO5-Re
**PDB code**	**6EUO**	**6EUR**	**6EXF**	**6EXH**	**6F9P**
Crystallization	23% PEG 3350,	25% PEG 4000,	25% PEG 4000,	25% PEG 4000,	24% PEG 3350,
	.2 M imidazole	.2 M imidazole	.2 M imidazole	.2 M imidazole	.2 M imidazole
	malate	malate	malate	malate	malate
	.15 M Li_2SO_4	.15 M Li_2SO_4	.15 M Li_2SO_4	.15 M Li_2SO_4	.15 M Li_2SO_4
	pH 7.0	pH 7.0	pH 7.0	pH 7.0	pH 7.0
Cryoprotectant	30% CM5[a],	20% glycerol,	20% glycerol,	20% glycerol,	30% CM5[a],
	25% PEG 3350,	27% PEG 4000,	27% PEG 4000,	27% PEG 4000,	26% PEG 3350,
	.1 M imidazole	.1 M imidazole	.1 M imidazole	.1 M imidazole	.2 M imidazole
	malate, pH 7,	malate, pH 7,	malate, pH 7,	malate, pH 7,	malate, pH 8
	.15 M Li_2SO_4	.15 M Li_2SO_4	.15 M Li_2SO_4	.15 M Li_2SO_4	.15 M Li_2SO_4
	20 sec. dip.	.001 M $FeSO_4$,	.001 M $FeSO_4$,	.001 M $FeSO_4$,	.2mM ReK_2Cl_6
		.05 M α-KG,	.02 M L-lysine,	.025 M α-KG,	20 min. soak.
		10 min. soak.	10 min. soak.	.02 M L-lysine,	
				10 min. soak.	
Data Collection					

Source	SOLEIL	SOLEIL	SOLEIL	SOLEIL	SOLEIL
Beamline	Proxima-2A	Proxima-1	Proxima-1	Proxima-2A	Proxima-1
Wavelength					
Space group	$P2_12_12_1$	$P2_12_12_1$	$P2_12_12_1$	$P2_12_12_1$	$P2_12_12_1$
Unit-cell (Å)	91.3 98.9 166.1	91.4 99.5 166.2	91.7 99.6 165.8	91.7 99.2 165.6	90.2 98.9 166.0
Z'	4	4	4	4	4
Resolution (Å)	50(2.36)-2.3	50(2.36)-2.3	50(2.00)-1.95	63.57(2.76)-2.6	50(2.46)-2.40
$CC_{1/2}$[b] (%)	99.8(54.7)	99.9(75.5)	99.9(7.9)	99.9(84.0)	99.6(81.4)
$\langle I/\sigma(I)\rangle$	13.3(1.16)	15.9(0.91)	13.9(0.18)	13.8(1.40)	20.5(1.5)
R_{meas}[c] (%)	12.5(204.6)	6.7(148.6)	6.7(611.8)	16.5(156.4)	12.7(185.4)
R_{factor}[c] (%)	11.8(183.2)	6.2(136.6)	6.2(548.7)	15.9(150.7)	12.5(178.9)
Complete (%)	99.9(99.9)	99.7(97.4)	99.6(96.0)	99.8(99.0)	100(99.9)
Multiplicity	9.2(5.0)	6.6(6.4)	7.1(4.9)	13.7(14.0)	27.8(14.4)
Anisotropy		STARANISO[e]	STARANISO[e]		
Resolution (Å)	-	50(2.36)-2.3	50(2.0)-1.95	-	-
$CC_{1/2}$[b] (%)	-	99.9(83.8)	99.9(22.0)	-	-
	-	18.11(1.79)	20.01(0.74)	-	-
R-meas[c] (%)	-	6.3(85.9)	5.7(173.2)	-	-
Complete (%)	-	87.1(38.9)	68.8(3.6)	-	-

Anisotropy resolution limits (Å)	-	2.52, 2.43, 2.17	2.36, 2.26, 1.95	-	-
Refinement	BUSTER[f]	BUSTER[f]	BUSTER[f]	BUSTER[f]	BUSTER[f]
Resolution (Å)	62.2(2.36)-2.3	33.7(2.36)-2.3	28.6(2.0)-1.95	38.7(2.67)-2.6	49.43(2.46)-2.4
No. of reflections	67467(4925)	59249(1847)	76433(289)	47189(3251)	58757 (4071)
R_{work} (%)	16.9(23.6)	17.4(21.7)	17.7(26.8)	17.7(24.11)	19.4(24.8)
R_{free} (%)	20.5(28.6)	21.3(24.7)	21.3(28.3)	22.6(25.86)	21.3(27.6)
RMSD bonds					
Lengths (Å)	0.010	0.010	0.010	0.010	0.010
Angles (°)	1.10	1.14	1.07	1.150	1.11
Ramachandran					
favored	98.0 %	97.0 %	98.0 %	96 %	98 %
outliers	0	2	3	3	1
Interfaces[g]					
A-B (Å^2) / CSS	1490.4 / 0.298	1476.7 / 0.383	1510.6 / 0.465	1479.7 / 0.466	
A-C (Å^2) / CSS	403.5 / 0.000	398.5 / 0.000	395.6 / 0.000	403.9 / 0.000	
B-C (Å^2) / CSS	608.4 / 0.000	610.5 / 0.000	601.3 / 0.000	627.0 / 0.000	
B-D (Å^2) / CSS	352.9 / 0.000	344.2 / 0.000	351.0 / 0.000	351.0 / 0.000	
C-D (Å^2) / CSS	1498.5 / 0.298	1487.4 / 0.383	1514.8 / 0.465	1504.0 / 0.466	

[a] CM5: 12.5 % di-ethylene glycol + 25 % ethylene glycol + 12.5 % MPD + 12.5 % glycerol + 12.5 % 1,2-propanediol + .0125 M NDSB 201[6]. [b] $CC_{1/2}$: Data quality correlation coefficient[2]. [c] Data collection statistics are from XDS[3] . [d] Anomalous data: F and F are treated as separate reflections. [e] STARANISO is a program from Global Phasing[7]. [f] BUSTER is a program from Global Phasing[4]. [g] Interface analyzed by PISA; CSS (Complexation Significance Score) indicates the significance of the interface for the assembly formation, with a maximal value of 1[5]. The area buried in the interface and the CSS score for the interface are reported.

Table S3. Flexible loops listed correspond to several consecutive residues with high B factor (>50). Unmodelled loops are listed in brackets.

Protein / Molecule	APO	α-KG	Lys	Products
**KDO1**				
A	30-36<br>161-168<br>234-235	34-37<br>106-110<br>116-120<br>160-172<br>218-220<br>234-235<br>249-252<br>269-271<br>275-284	34-37<br>42-55<br>93-96<br>102-120<br>124-130<br>147-149<br>160-169<br>213-253<br>260-262<br>269-285<br>322-325	34-37<br>42-55<br>93-96<br>102-120<br>147-149<br>160-170<br>213-252<br>257-261<br>268-284
B	30-37<br>159-177<br>231-236<br>238-245<br>248-252<br>269-284<br>316-324	33-37<br>93-96<br>105-108<br>115-119<br>145-149<br>156-159<br>(160-169)<br>170-178<br>200-203<br>213-220<br>225-252<br>267-297<br>316-328	35-55<br>94-98<br>100-130<br>145-178<br>160-1641195-205<br>210-220<br>223-253<br>267-303<br>316-330<br>338-341<br>354-358	33-51<br>102-121<br>124-130<br>145-180<br>196-205<br>214-220<br>223-253<br>267-303<br>314-330<br>355-358
C	31-36<br>116-119<br>159-172	33-37<br>105-120<br>159-174<br>231-235<br>269-271<br>321-325	33-37<br>40-62<br>77-81<br>102-130<br>145-174<br>217-252<br>269-285<br>320-327	34-37<br>41-59<br>102-130<br>147-149<br>154-176<br>215-252<br>269-284<br>318-327

D	30-36<br>115-117<br>158-160<br>(161-167)<br>168-176<br>231-243<br>248-252<br>268-284<br>316-324	33-37<br>114-119<br>147-149<br>156-159<br>(160-173)<br>174-178<br>199-202<br>230-252<br>268-298<br>316-328	35-51<br>76-81<br>93-95<br>102-130<br>145-151<br>154-180<br>196-205<br>214-220<br>226-252<br>268-303<br>314-330	33-51<br>76-81<br>93-96<br>102-121<br>124-130<br>145-151<br>154-178<br>196-205<br>215-220<br>223-252<br>268-303<br>316-330
**KDO5**				
A	17-87<br>98-123<br>142-146<br>155-175<br>195-205<br>208-230<br>(231-240)<br>241-245<br>258-299<br>313-338<br>348-372	19-21<br>35-56<br>98-123<br>155-174<br>197-199<br>226-229<br>(230-240)<br>241-244<br>265-275<br>293-295<br>314-335<br>353-356<br>367-372	19-21<br>29-60<br>67-75<br>98-123<br>155-174<br>195-202<br>228-230<br>(231-240)<br>241-244<br>263-275<br>292-299<br>313-338<br>353-355<br>367-371	17-231<br>(232-239)<br>240-372
B	19-123<br>140-<br>158-184<br>189-230<br>(231-240)<br>241-321<br>(322-330)<br>331-340<br>353-372	20-24<br>32-36<br>51-60<br>70-72<br>85-87<br>98-123<br>140-179<br>193-205<br>210-229<br>(230-241)<br>242-247<br>252-254<br>258-296<br>(315-327)<br>328-338<br>362-372	19-40<br>47-60<br>67-75<br>98-123<br>140-154<br>(155-167)<br>168-183<br>191-230<br>(231-241)<br>242-248<br>251-254<br>258-299<br>313-315<br>(316-330)<br>331-338<br>362-372	19-229<br>(230-241)<br>242-321<br>(322-327)<br>328-372

C	13-60<br>67-126<br>140-232<br>(233-237)<br>238-338<br>343-371	15-29<br>82-89<br>98-123<br>140-148<br>153-185<br>193-229<br>(230-241)<br>242-299<br>308-338<br>353-355<br>361-372	14-38<br>47-60<br>70-89<br>98-123<br>142-148<br>152-182<br>191-233<br>(234-240)<br>241-299<br>309-338<br>353-355<br>361-372	15-233<br>(234-238)<br>239-372
D	18-80<br>87-89<br>92-185<br>189-230<br>(231-241)<br>242-372	18-21<br>27-59<br>95-125<br>140-181<br>191-204<br>222-230<br>(231-241)<br>242-246<br>260-301<br>312-338<br>347-372	17-20<br>27-59<br>95-125<br>140-181<br>191-205<br>222-230<br>(231-240)<br>241-246<br>258-301<br>312-338<br>346-372	17-234<br>(235-240)<br>241-372

Table S4. Ligand-KDO5 interactions.

Structure	Interaction[a]	Ligand atom	Protein atom	Distance (Å)
KDO5 -αKG (monomer A)	H-bond	O1	HIS 312-N2	3.31
	H-bond	O1	GLU 178-O2	2.72
	H-bond	O1	ARG 338-NH1	2.91
	H-bond	O3	TYR 193-OH	2.97
	H-bond	O3	ARG 334-NH2	3.07
	H-bond	O3	H_2O-555	3.07
	H-bond	O4	ARG 334-NH1	2.77
	H-bond	O4	ARG 334-NH2	3.27
	H-bond	O4	H_2O-543	2.71
	van der Waals	C3	LEU 173-CD2	3.72
KDO5 Lys (monomer C)	H-bond	N	GLN 144-O1	2.79
	H-bond	OXT	SER 167-N	3.72
	H-bond	OXT	SER 167-O_γ	3.52
	H-bond	Nε	HIS 176-NE2	3.05
	H-bond	Nε	H_2O-578	2.81
	H-bond	OXT	H_2O-619	3.00
	H-bond	OXT	H_2O-524	2.70

	Salt bridge	O	ARG 145-NH2	3.56
	Salt bridge	O	ARG 338-NH1	3.24
	Salt bridge	O	ARG 338-NH2	3.81
	Salt bridge	OXT	ARG 145-NH2	2.97
	van der Waals	Ca	THR 165-OG1	3.4
	van der Waals	O	GLY 166-Ca	3.4
KDO5 products[b] (monomer D)	H-bond	SIN-O1	HIS 312-N2	3.21
	H-bond	SIN-O2	ARG 338-NH1	3.08
	H-bond	SIN-O2	GLU 178-OE2	3.01
	H-bond	SIN-O3	ARG 334-NH1	3.36
	H-bond	SIN-O3	ARG 334-NH2	3.30
	H-bond	SIN-O4	TYR 193-OH	3.31
	H-bond	LYO-N	GLN 144-O1	2.85
	H-bond	LYO-Og	GLU 178-O1	3.64
	H-bond	LYO-Og	ASP 230-O2	3.63
	H-bond	LYO-Nε	ASP 260-O2	2.78
	H-bond	LYO-O	THR 165-O1	2.84

H-bond	LYO-O	H_2O-530	2.78
Salt bridge	LYO-N	GLU 178-O1	3.83
Salt bridge	LYO-N	ASP 230-O2	3.40
Salt bridge	LYO-OXT	ARG 338-NH1	2.78
van der Waals	LYO-CD	LEU 173-CD1	3.81
van der Waals	SIN-C3	LEU 173-CD2	3.81

[a] The interactions were calculated using a PDBe-PISA server[5]. [b] The PDB ligands codes “SIN” and “LYO” have been used here to designate succinate and 4-hydroxy-lysine.

Table S5. Ligand-KDO1 interactions.

Structure	Interaction[a]	Ligand atom	Protein atom	Distance (Å)
KDO1 α-KG (monomer B)	H-bond	O1	HIS 314-N2	3.17
	H-bond	O1	ARG 332-NH1	2.69
	H-bond	O1	GLU 180-OE1	2.75
	H-bond	O3	ARG 328-NH2	2.96
	H-bond	O3	THR 204-OG1	2.71
	H-bond	O4	ARG 316-NE	3.75
	H-bond	O4	ARG 328-NH1	2.79
	H-bond	O4	ARG 328-NH2	3.23
	H-bond	O4	H_2O-543	2.70
KDO1 Lys (monomer C)	H-bond	O	SER 168-Og	3.21
	H-bond	OXT	SER 168-N	3.28
	H-bond	OXT	SER 236-Og	3.33
	H-bond	OXT	H_2O-547	2.70
	H-bond	OXT	H_2O-638	3.10
	H-bond	N	H_2O-502	2.76
	H-bond	Nε	H_2O-513	2.75

	Salt bridge	N	GLU 180-O1	3.67
	Salt bridge	N	GLU 180-O2	2.85
	Salt bridge	N	ASP 234-O2	3.91
	Salt bridge	Nε	ASP 268-O1	3.39
	Salt bridge	Nε	ASP 268-O2	2.70
	Salt bridge	Nε	ASP 234-O1	2.92
	Salt bridge	O	ARG 332-NH1	3.05
	Salt bridge	O	ARG 332-NH2	3.03
KDO1 products[b] (monomer B)	H-bond	SIN-O1	ARG 332-NH1	3.23
	H-bond	SIN-O2	HIS 314-N2	3.44
	H-bond	SIN-O3	ARG 328-NH2	3.09
	H-bond	SIN-O3	THR 204-O1	2.99
	H-bond	SIN-O4	ARG 328-NH1	2.95
	H-bond	CUW-N	SER 236-Og	2.90
	H-bond	CUW-O	SER 168-Og	3.40

	CUW-O	SER-236-Og	3.30
H-bond	CUW-OXT	ARG 332-NH1	3.00
H-bond	CUW-OXT	ARG 332-NH2	3.00
H-bond	CUW-Ob	GLU 180-O2	3.20
H-bond	CUW-N	ASP 234-O1	3.00
H-bond	CUW-Nε	ASP 268-O2	2.80
H-bond	CUW-Ob	H_2O-511	2.70
H-bond	CUW-Ob	H_2O-536	2.70
H-bond	CUW-Nε	H_2O-562	2.90
van der Waals	SIN-C2	LEU 193-CD1	3.68

[a] The interactions were calculated using PDBe-PISA server[5]. [b] The PDB ligands codes "SIN" and "CUW" have been used here to designate succinate and 3-hydroxy-lysine.

Table S6: Inventory of the InterPro families that contain α-KG-dependent dioxygenases and description of the associated α-ketoglutarate dioxygenases when their actives on amino acids or derivatives. The majority of them have in common the utilization of a Fe(IV)-oxo intermediate to initiate diverse oxidative transformations[8]. The mechanism involves a metallocenter bound on one face by three protein side chains forming a high valent iron:oxygen atom intermediate[9].

Enzymes	Substrates	UniProtKB	Organisms	PDBs	Seq. identity* with KDO1 and KDO5	CATH classification
1. α-ketoglutarate dioxygenases actives on free amino acids or their derivative						
1.1.Clavaminate synthase-like (IPR014503)						
MppO	L-enduracididine	Q643C1	*Streptomyces hygroscopicus*	*N.A.*	30% with KDO1<br>25% with KDO5	*N.A*
VioC	Alpha-ketoglutarate-dependent L-arginine hydroxylase	Q6WZB0	*Streptomyces vinaceus*	2WBO<br>6ALM	26% with KDO1<br>19% with KDO5	**3.60.130** Double-stranded beta-helix<br>**3.60.130.10** Clavaminate synthase-like<br>Domain 2wbqA00
AsnO	L-asparagine	Q9Z4Z5	Streptomyces coelicolor A3(2)	2OG5-7	35% with KDO1<br>27% with KDO5	**3.60.130** Double-stranded beta-helix<br>**3.60.130.10** Clavaminate synthase-like<br>Domain 2og5A00
OrfP	L-arginine	G9MBV2	*Streptomyces lavendulae subsp. lavendulae*	4NE0, 4M2M, 4M2K, 4M2J, 4M2I, 4M2F, 4M2G, 4M2E, 4M2C, 4M27,	30% with KDO1<br>27% with KDO5	**3.60.130** Double-stranded beta-helix<br>**3.60.130.10** Clavaminate synthase-like<br>Domain 2wbqA00

				4M26, 4M25, 4M23		
KDO1	L-lysine (Hydroxylase in C3)	C7QJ42	Catenulispora acidiphila (strain DSM 44928)	*this study*	20 % with KDO5	**3.60.130** Double-stranded beta-helix<br><br>**3.60.130.10** Clavaminate synthase-like<br><br>Domain 2og5A00
KDO5	L-lysine (Hydroxylase in C4)	J3BZS6	*Flavobacterium sp. (strain CF136)*	*this study*	20 % with KDO1	**3.60.130** Double-stranded beta-helix<br><br>**3.60.130.10** Clavaminate synthase-like<br><br>Domain 2wbqA00
ODO		C7Q942		*N.A.*		*N.A.*
CAS1	Clavaminate	Q05581	*Streptomyces clavuligerus*	*N.A.*	30% with KDO1 30% of 60% of the the sequence with KDO5	*N.A.*
CAS2	Clavaminate	Q05582	*Streptomyces clavuligerus*	*N.A.*	34% with KDO1 23 with KDO5	*N.A.*
NapI	L-arginine	S4TL40	*Streptomyces lusitanus*	6DAW	29% with KDO1 25% with KDO5	**3.60.130.10** Clavaminate synthase-like
1.2. Phytanoyl-CoA dioxygenase (IPR008775)						
GriE	L-Leucine (and various other aliphatic amino acids)<br><br>Involved in biosynthesis of	A0A0E3U RV8	Streptomyces muensis	5NCI	13% with KDO1 11% with KDO5	**2.60.120** Jelly Rolls<br><br>**2.60.120.620** q2cbj1_9rhob like domain

	4-methylproline					Domain 4j25F00
Unclassified in InterPro (on the whole sequence°						
P3H	Proline 3-hydroxylation (type II)	O09345	*Streptomyces sp*	1E5S	No sequence identity	**2.60.120.330**<br><br>Domain 1e5rB01
AspH	Aspartate β-hydroxylase isoform	Q12797	*Homo sapiens*	5APA	No sequence identity	**2.60.120.330**<br><br>Domain 1e5rB01
SadA	stereoselectively catalyzes the C3-hydroxylation of *N*-substituted branched-chain or aromatic L-amino acids, especially *N*-succinyl-L-leucine,	Q0B2N4	*Burkholderia ambifaria*	3W21	14% with KDO1<br>12% with KDO5	**2.60.120** Jelly Rolls<br><br>**2.60.120.590** Clavaminate synthase-like domains<br><br>Domain 3s57A00
1.3. 2OG-Fe dioxygenase (IPR018724)						
IDO	L-isoleucine and many other substrates (Smirnov et al. 2012)(L-norvaline, L-norleucine and L-allo-isoleucine, as well as the sulfoxidation of L-methionine, L-ethionine, S-methyl-L-cysteine, S-ethyl-L-cysteine, and S-allyl-L-cysteine)	E2GIN1	*Bacillus thuringiensis*	*N.A.*	No sequence identity	*N.A.*

MpDO	Unknown function<br><br>Hydroxyle methyl tert-butyl ether (MTBE)(Xu et al. 2014)	A2SJH7	*Methylobium petroleophilum*	3PL0	15% with KDO1<br>9% with KDO5	**2.60.120 Jelly Rolls**<br><br>**2.60.120.620**<br>q2cbj1_9rhob like domain<br><br>Domain 4j25F00
2. ɑ-ketoglutarate dioxygenases actives on other compounds						
2.1. InterPro family						
Putative 2OG-Fe(II) oxygenase (IPR030975)						
Alpha-ketoglutarate-dependent dioxygenase AlkB-like superfamily (IPR037151)						
2.2 InterPro domains						
Aspartyl/Asparaginyl beta-hydroxylase domain (IPR007803)						
TauD/TfdA-like domain (IPR003819), in which KtzO (Uniprot ID: A8CF77, no structure), Ktzp (A8CF76, no structure), SyrP (Q4ZT71, no structure) proteins are included. These enzymes catalyze the hydroxylation of Peptidyl-Carrier-Protein amino acids[10]. These enzymes do not share sequence identity with enzymes from the CSL family.						
2OGFeDO, oxygenase domain (IPR024779)						
Alpha-ketoglutarate-dependent dioxygenase alkB homologue 4 (IPR032857)						
Alpha-ketoglutarate-dependent dioxygenase AlkB-like (IPR027450)						
Domain : Oxoglutarate/iron-dependent dioxygenase (IPR005123)						

* with blastp not having any prior structural alignment

Table S7 | Representation of the variability in term of sequence and length for the flexible lid in the CSL family

ASMC Group	Logo of the lid (from start of the lid to minimum length)	Length min-max
G1	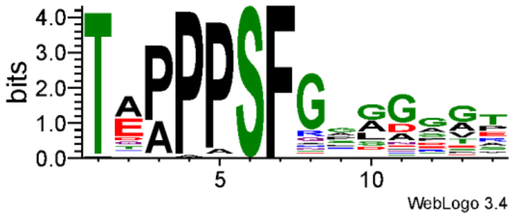	*15-20*
G2	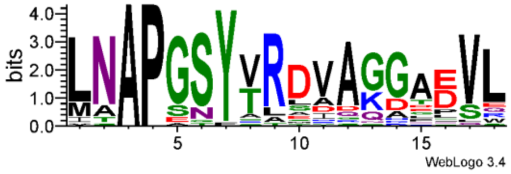	*19-24*
G3	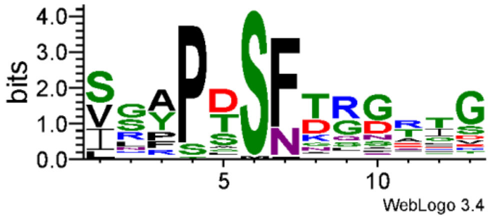	*14-22*
G4	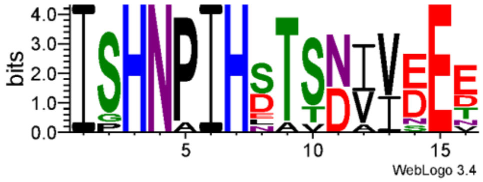	*17-17*
G5	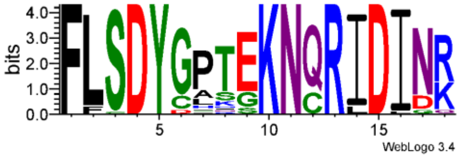	*19-19*
G6	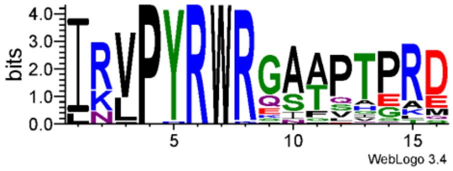	*17-20*

G7	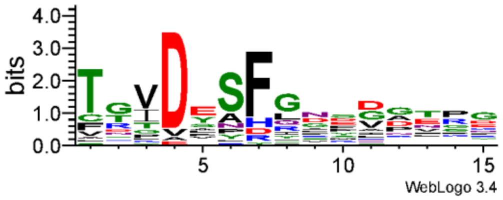	*16-24*
G8	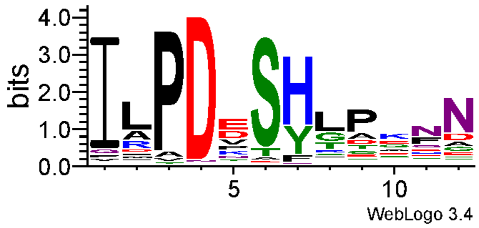	*13-71*
G9	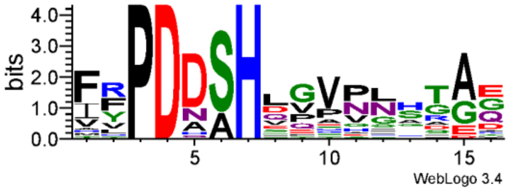	*17-36*
G10	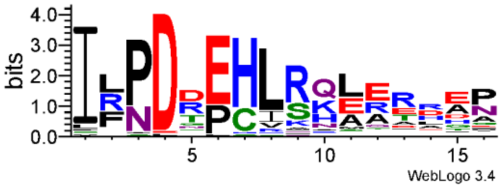	*17-33*
G11	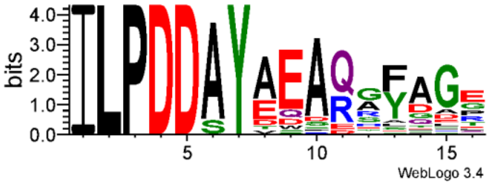	*17-20*
G12	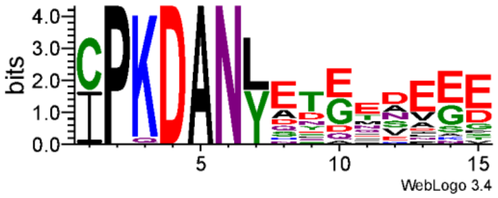	*16-18*

Supplementary figures

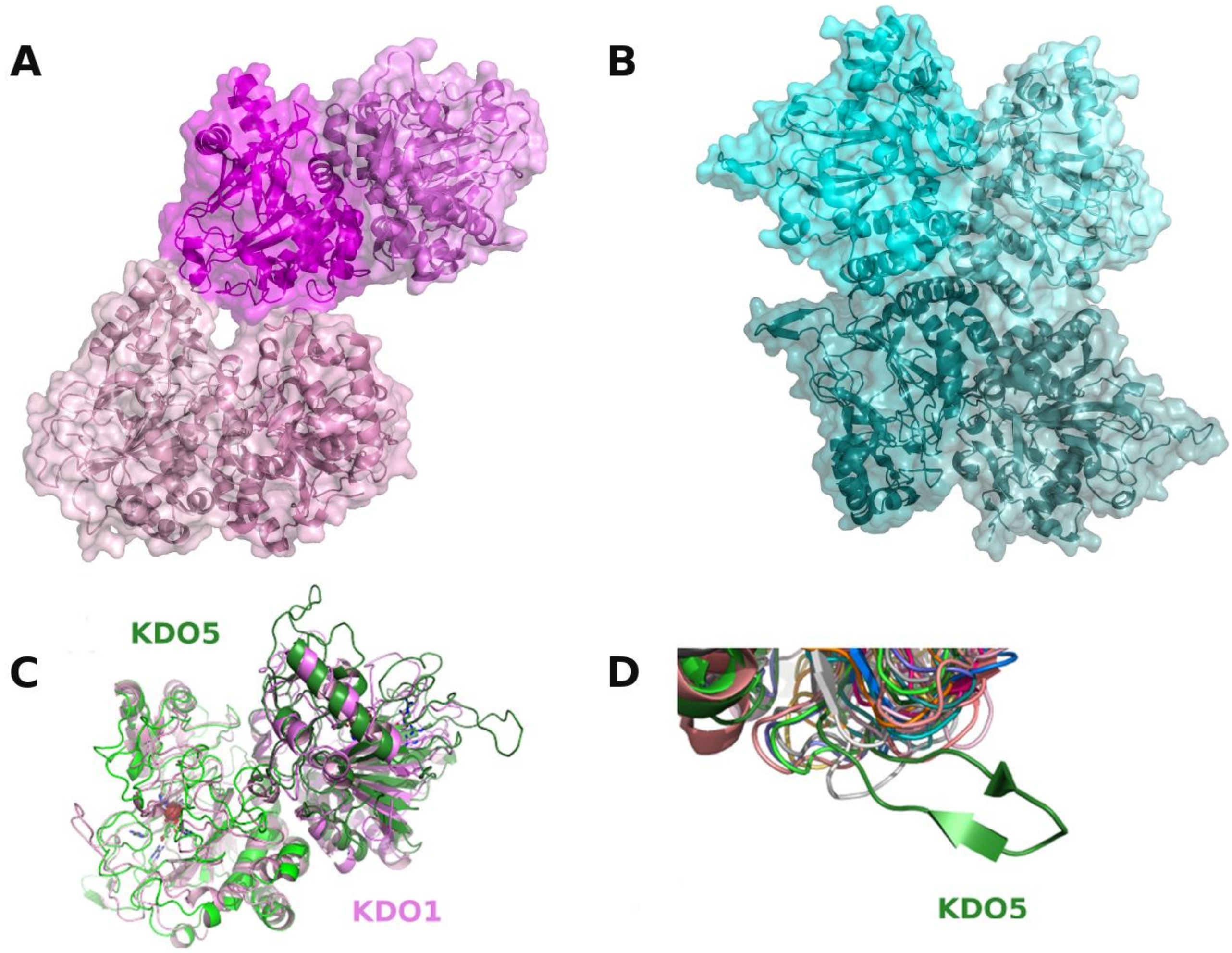

Figure S1 | Comparison of the crystal structures of KDO1 and KDO5. (**A, B**) Asymmetric unit of, respectively, KDO1 and KDO5 crystals, characterized by four molecules (A, B, C and D), and assembled as a dimer of dimers. (**C**) Superimposition of dimers for KDO1 and KDO5 in cartoon representation showing helices and sheet. (**D**) Superimposition of monomers of KDO1, KDO5 and other iron-ketoglutarate dependent dioxygenases from **Table S3** showing that only KDO5 has a long loop in the 321-334 region.

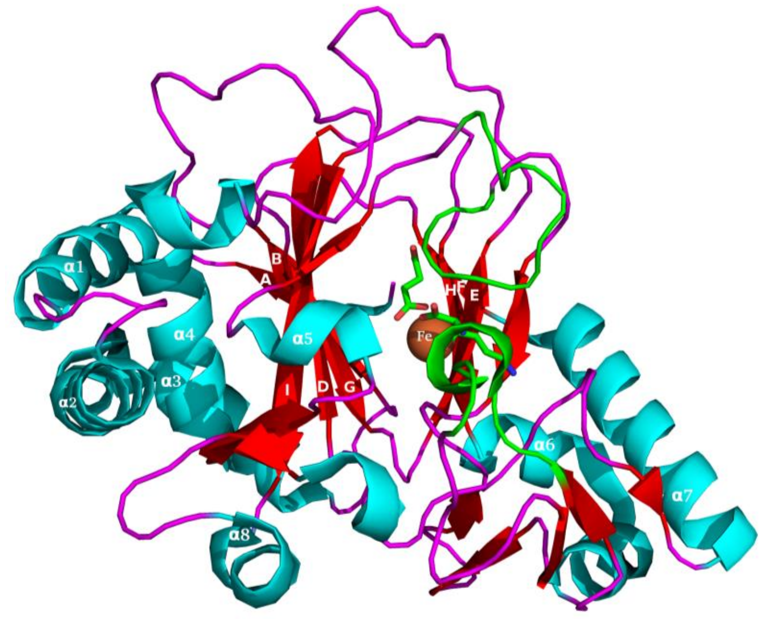

Figure S2 | Overall structure of the KDO1-Fe(II)-succinate-3-hydroxy-lysine complex (monomer B). Helices, β-strands and loops are shown in cyan, red and pink respectively. The lid region and the adjacent loop are shown in green, the bound Fe(II) in orange, and the succinate and the 3-hydroxy-lysine in green.

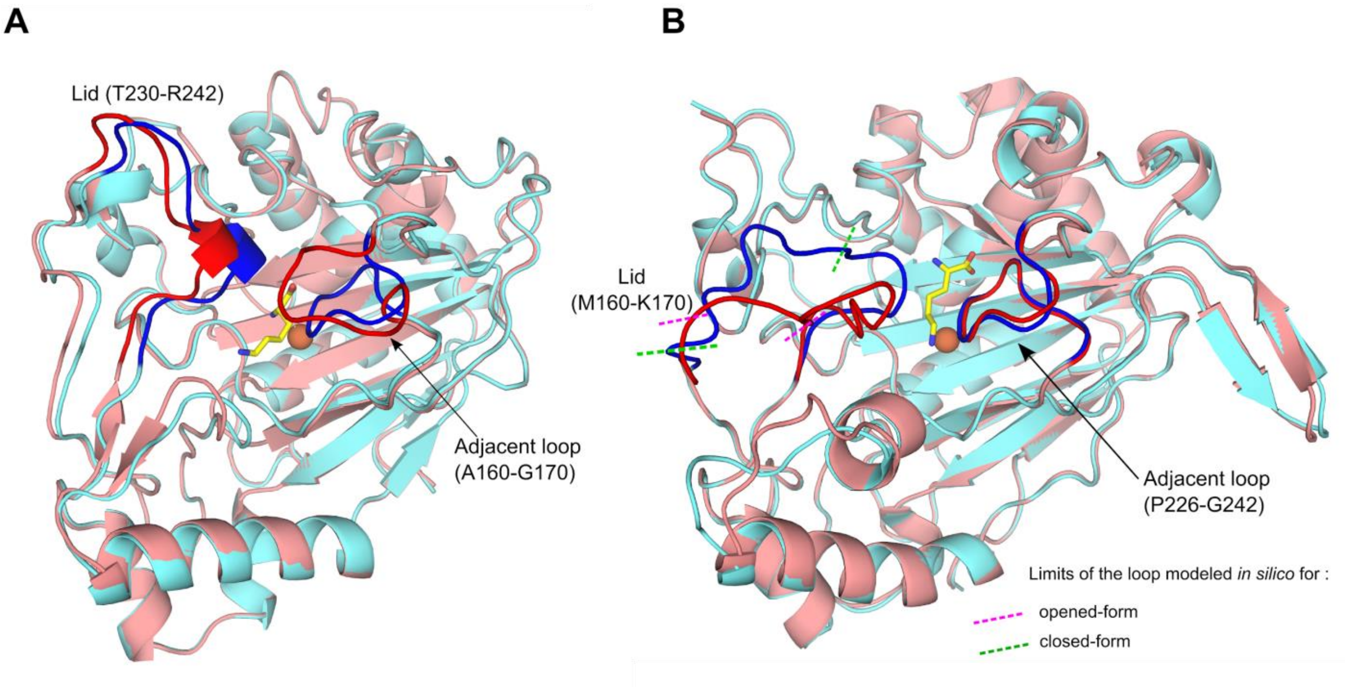

Figure S3 | Adjacent loop and flexible lid in KDO1 and KDO5. (**A**) Superimposition of KDO1 apo-form (salmon) and KDO1 bound to lysine (yellow for substrate and cyan for protein) from monomer A. The two important loops named “lid” and “adjacent loop” are colored more intensely. On the apo-form, the adjacent loop is in closed form, while in the presence of lysine the adjacent loop is in an opened form. (**B**) Superimposition of KDO5 apo-form (salmon) and bound to lysine (in yellow for substrate and cyan for protein) from monomer C. The lid is not resolved in any crystallographic structures of KDO5 but was modeled using Modeller software. Regions from crystallographic data are colored more intensely while the modeled regions are delimited with pink and green dashed lines for apo- (*i.e.* opened) and bound (*i.e.* bound) form respectively. The flexible lid is in an opened-form in the absence of substrates while it is in closed form when bound to lysine.

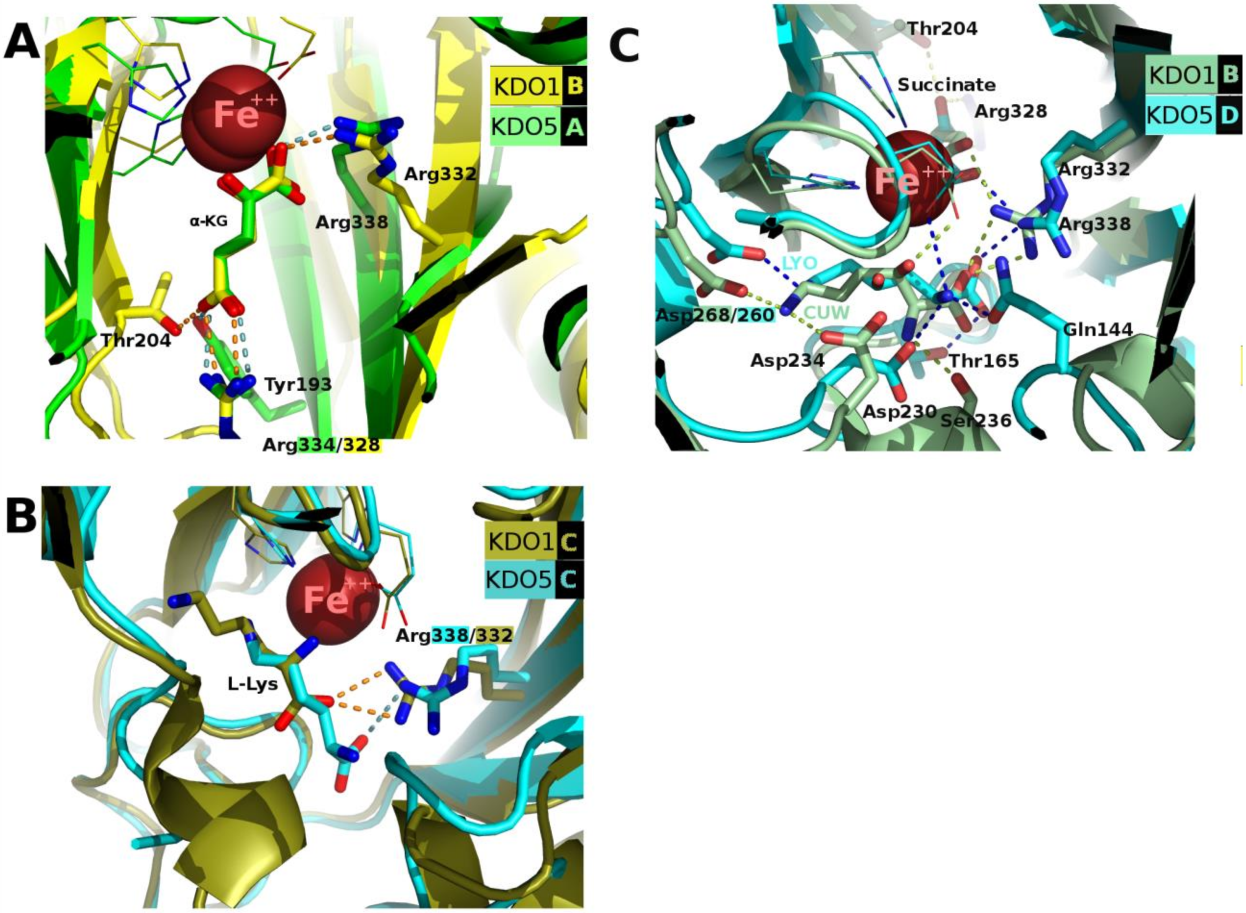

Figure S4 | Superimposition of KDO1 and KDO5 binding sites with the substrates bound. (**A**) KDO1 monomer B (yellow) and KDO5 monomer A (green) in complex with α-KG. (**B**) KDO1 (olive) and KDO5 (cyan) in complex with lysine (Both monomer C). (**C**) KDO1 monomer B (pale green) and KDO5 monomer D (cyan) in complex with respectively (3*S*)-3-hydroxy-L-lysine and (4*R*)-4-hydroxy-L-lysine.

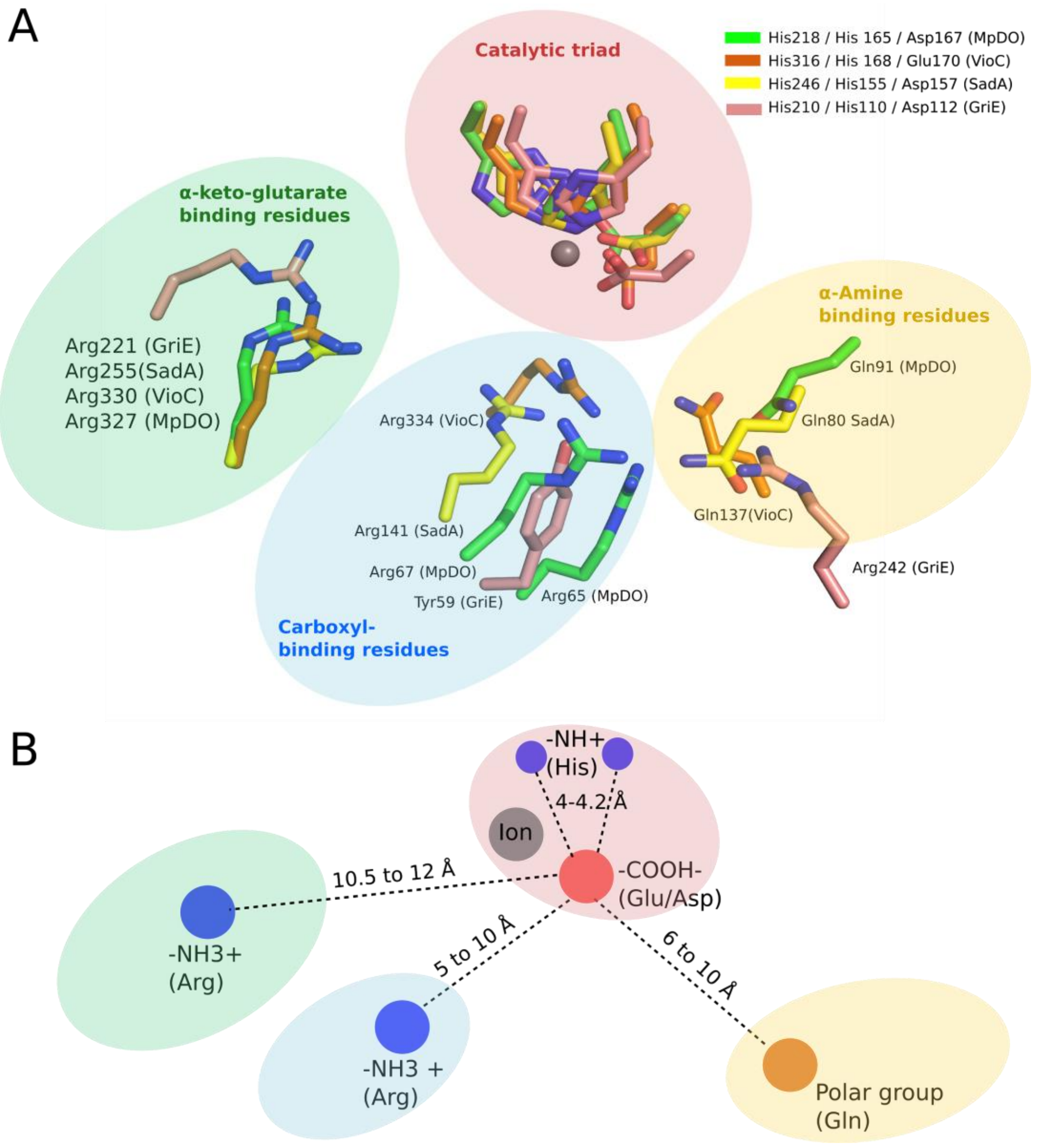

Figure S5 | Spatial arrangement of important residues from InterPro families in αKAOs that transform amino acids. (**A**) Superposition of active site residues from αKAOs that catalyze the transformation of free L-amino acids (or derivatives) but have different folds (**see Table S6**). Residues of the catalytic triad have been superimposed using Pair fit function of Pymol: in orange for VioC (PDB code 2WBP)[1]; in green for MpDO (3PL0)[11]; in pink for GriE (5NCI)[12]; and in yellow for SadA (3W21). These αKAOs show conserved structural features shared in the majority of αKAOs. Firstly, the Fe(II) is coordinated to two His and to a carboxylate from either a Glu or an Asp, with the exception of the halogenases, forming the so-called 2-His-1-carboxylate facial triad[13,14]. Secondly, the carboxylate of α-KG is stabilized through a salt bridge with an Arg (in the majority of cases) or a Lys[15,16]. MpDO structure was resolved without substrate but authors claim the methionine substrate is stabilized by Gln91 and Arg67 after having run docking simulation. The crystallographic structure of SadA was resolved only in presence of α-KG. Its C-5 carboxylate forms salt bridges with Arg255 and Arg141. We found that Arg141 occupies the same 3D-position than Arg334 in VioC (shown to binds the carboxylate of the

amino acid substrate) and Arg67 in MpDO. Because of his spatial arrangement, Arg141 we hypothesized that this residues can interacts with the carboxylate group of *N*-substituted aromatic substrate it transforms. It should be noted that the position of the carboxyl (Arg242) and α-amine (Tyr59) binding residues is inverted for GriE. For clarity, only one ion for the 4 proteins has been represented. (**B**) Spatial arrangement of functional groups indicates an active site constellation, defined by the distance between the functional groups. The distance does not include ions, as 3D-motif generally only includes coordinates of residues or functional groups.

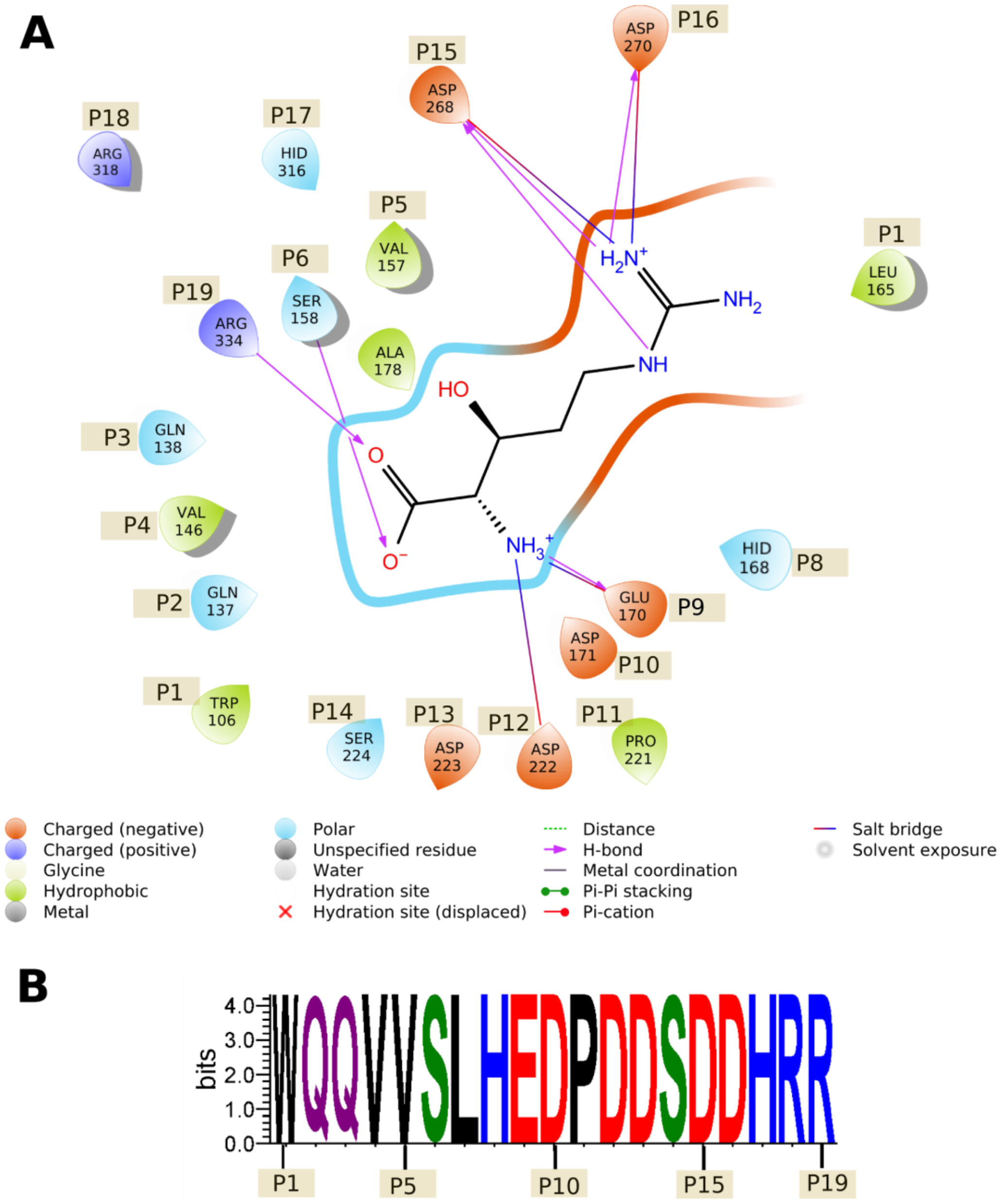

Figure S6 | Corresponding between position of residues in active site pocket and in logo sequence for VioC (**A**) Ligand-protein interaction from Maestro software (Schrödinger Release 2018-2: Maestro, Schrödinger, LLC, New York, NY, 2018) complexed with (2S,3S)-hydroxyarginine (PDB code: 2WBQ). Positions in the logo are indicated in beige rectangle. (**B**) Logo sequence of VioC generated by Weblogo[17] .

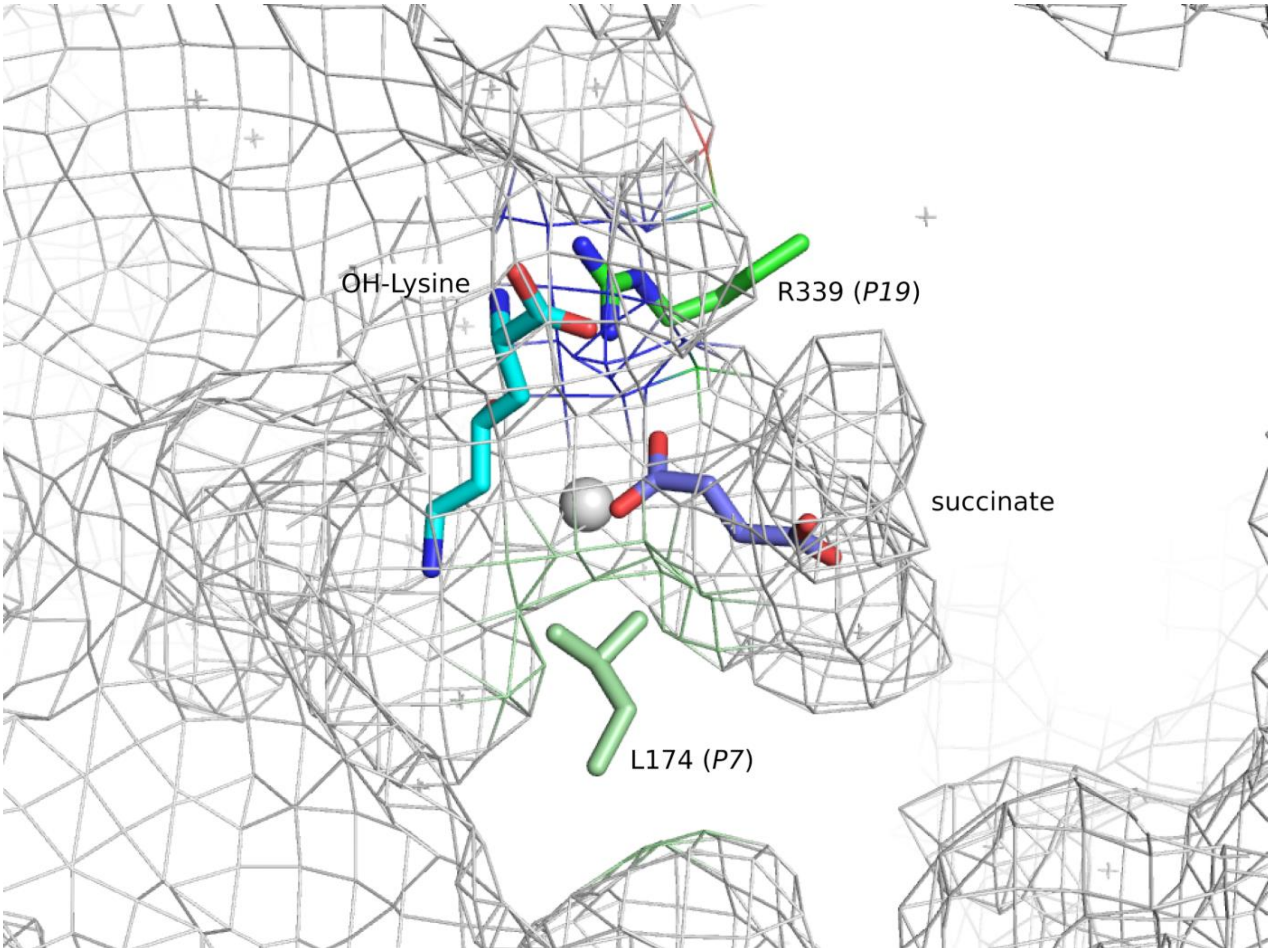

Figure S7 | Mesh representation of the pockets hosting succinate and hydroxy-lysine and delimited by Leu and Arg, respectively in *P7* and in *P19* of the logo. Position of hydroxy-lysine (represented by the cyan bar) and succinate (purple bar) were extracted from monomer D of KDO5. Note that the hydroxyl group of OH-Lys is hidden in this picture. The two pockets enclosing hydroxy-lysine and succinate are separated by the two-facing residues Leu174 and Arg339, that are conserved at 89 and 93% respectively in the CSL family. The impact of these two residue's side-chain on the pocket separation is indicated in light green for Leu174 and in bright green for Arg339. The sphere represents the iron.

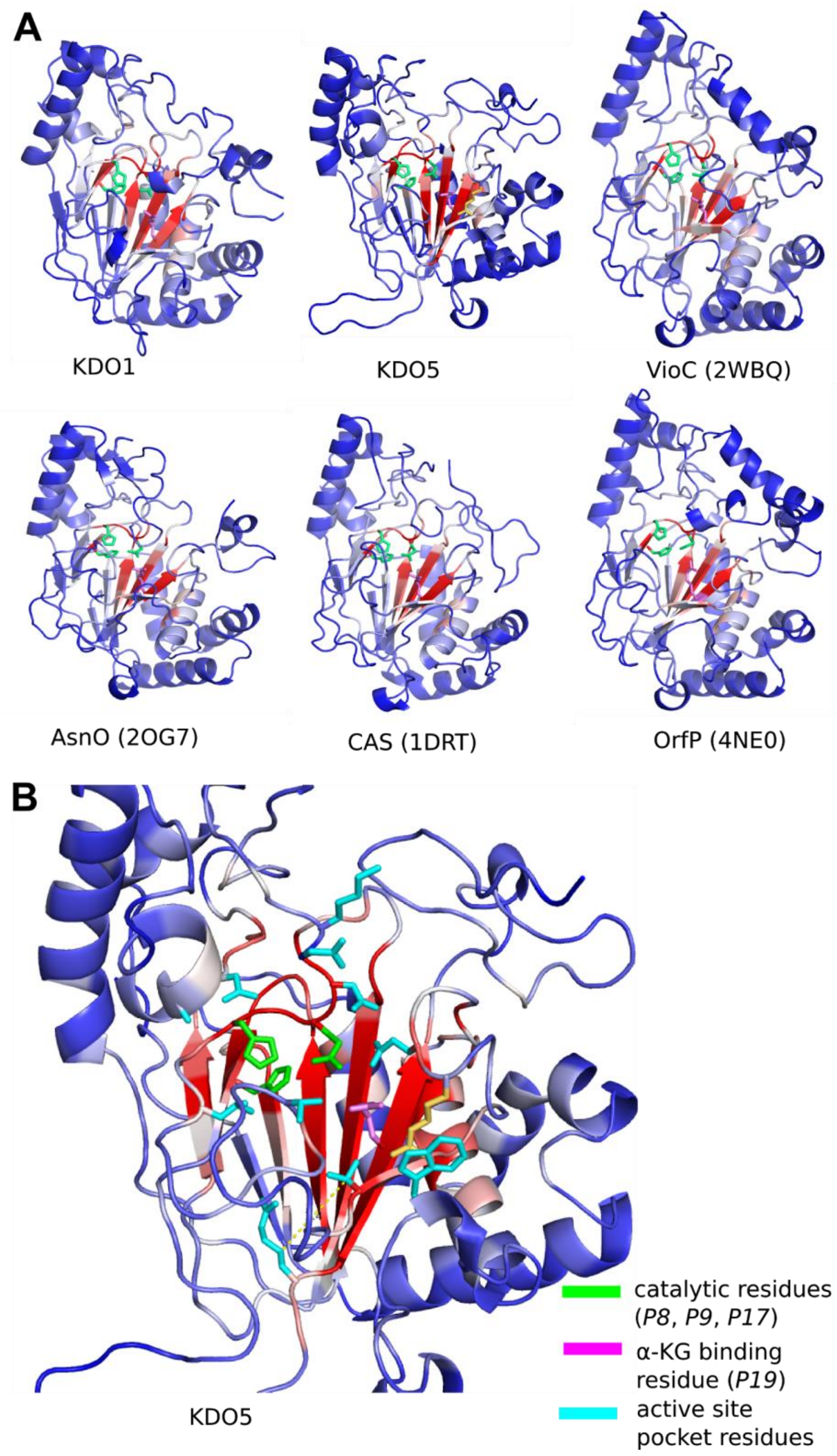

Figure S8 | Force constant measurements mapped on CSL crystal structures. Program used is ProPHet, with default parameters, which combines a coarse-grain / elastic network (ENM) protein model and a Brownian Dynamics algorithm to compute local rigidity of proteins at the residue level[18-20].

Cartoon representation of proteins with backbone colored on a spectrum ranging from red to white, to blue for the most, average and least rigid residues. **(A)** KDO1, KDO5, VioC, AsnO, CAS and OrfP show the same profile of rigidity. The residues implied in the catalysis and in α-KG binding are positioned in the rigid region of the proteins. PDB codes of the structures are indicated in brackets. **(B)** Details of KDO5's rigidity highlights that the side chain of Arg145 (*P3*) responsible for reaction's regioselectivity, is interacting with helix I, which is highly rigid. Other residues such as the catalytic triad (in green), and Arg in *P19* (in purple), are involved in rigid regions. By contrast, other residues of the pocket (in cyan) are not involved in rigid regions.

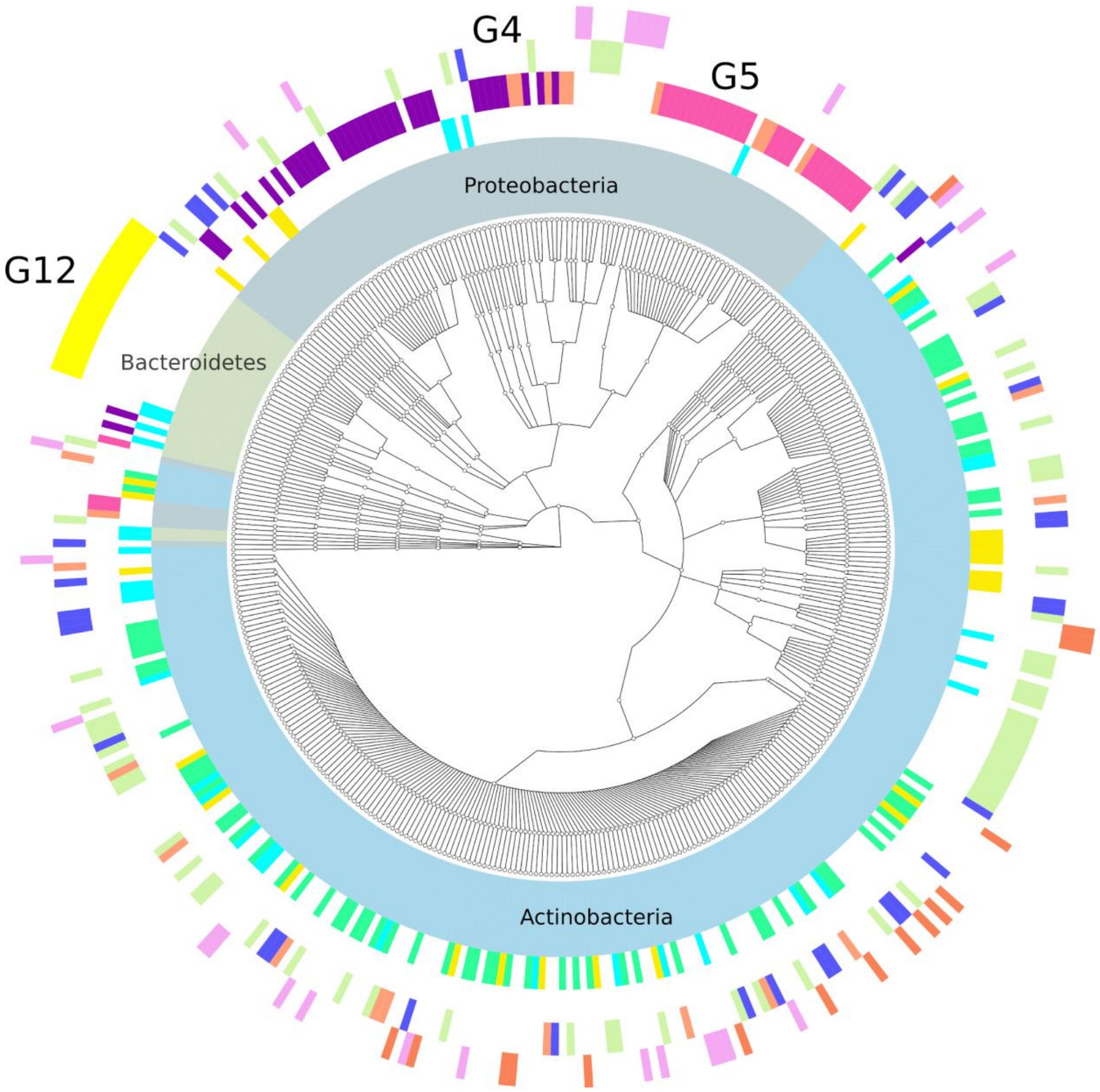

Figure S9 | Taxonomic tree from kingdom to species with ASMC group mapped. ASMC G1 to G12 groups have different colors: G1 to G3 on Ring 2 are in cyan, yellow and green respectively. G4 to G6 on Ring 3 are in orange, pink and purple respectively. G7 to G9 on Ring 4 are in light green, blue and orange respectively. G10 to G12 on Ring 5 are in pink, orange and yellow respectively. First ring highlights the phylum belonging. The G12 group that gathers KDO5 homologs is only found in two classes of bacteria (Chitinophagia and Flavobacteria). Group G5, expected to hydroxylate the substrate in C4, is detected in the Proteobacteria phylum only. Group G6, for which an arginine is positioned on the flexible loop, is present only in γ-proteobacteria. The oldest taxonomic levels, such as Actinobacteria phylum, a great diversity of CSL dioxygenases with more common features can be found.

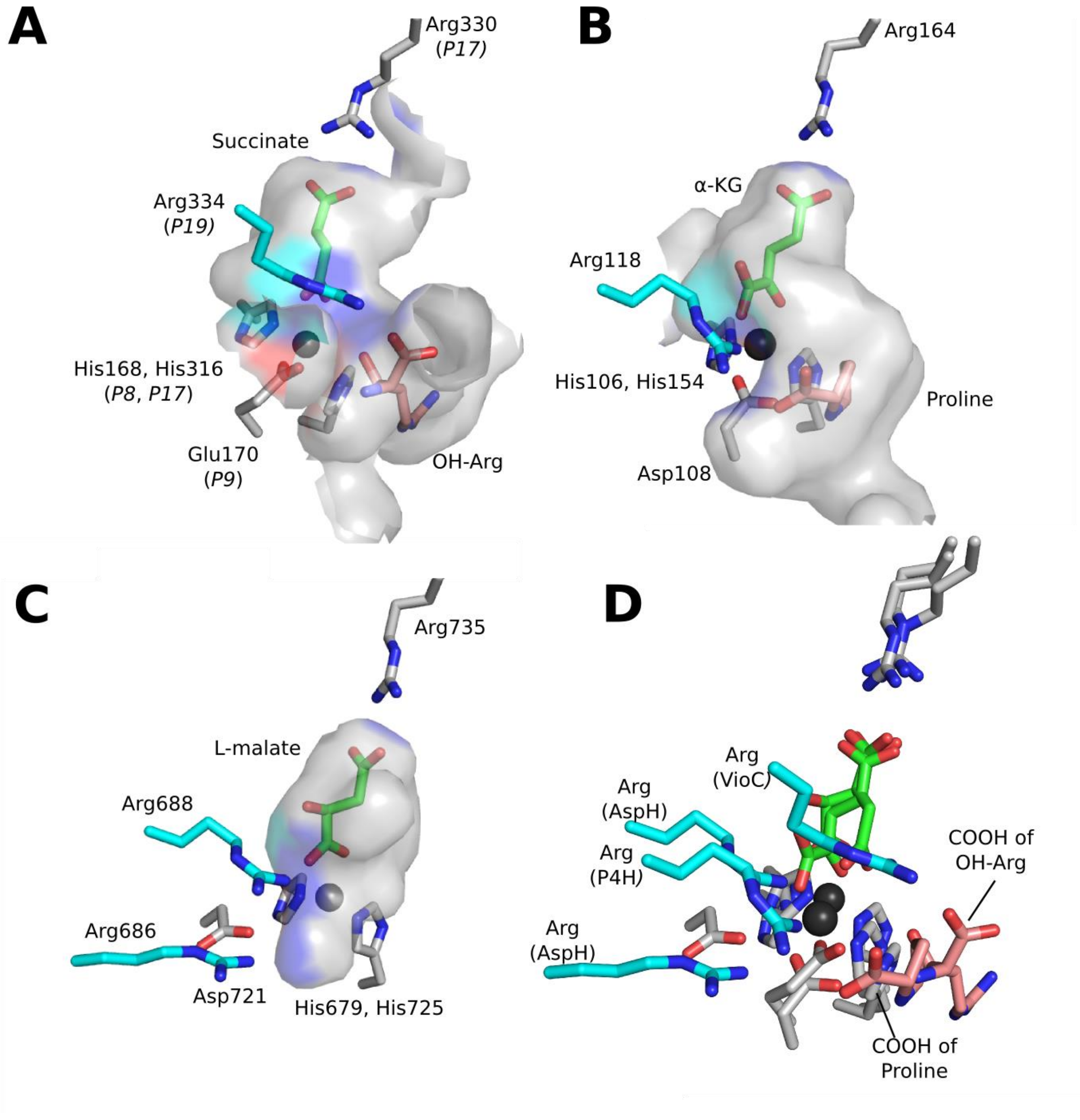

Figure S10 | Binding pocket comparison between VioC with α-KG dependent dioxygenases hydroxylating non free amino acids. Enzymes have been aligned using a Pair fit script in Pymol, aligning the catalytic triad and α-KG (in green) binding Arg (all represented in the gray stick). The volume of the binding pocket is represented in transparent gray. Residues interacting with the carboxylate of the substrate/product (in salmon) are colored in cyan. (**A**) Arginine dioxygenase VioC (PDB code 2WBP). (**B**) L-proline cis-4-hydroxylase complexed with L-proline, P4H (4P7W). Proline 3-hydroxylase (type II) (1E5S) is not shown on the figure as its binding pocket is very similar to P4H. (**C**) Aspartate β-hydroxylase isoform, AspH (5APA). α-KG is present but no crystal structure is available

with the substrate in the pocket, thus the substrate is absent from the figure. (**D**) Superposition of catalytic and binding residues of VioC, P4H and AspH. Orientation of the substrate is different between VioC and P4H/AspH, their carboxylate group being distant by 4.5 Å. Thus the binding pocket of substrate/product differs substantially[15,21,22].

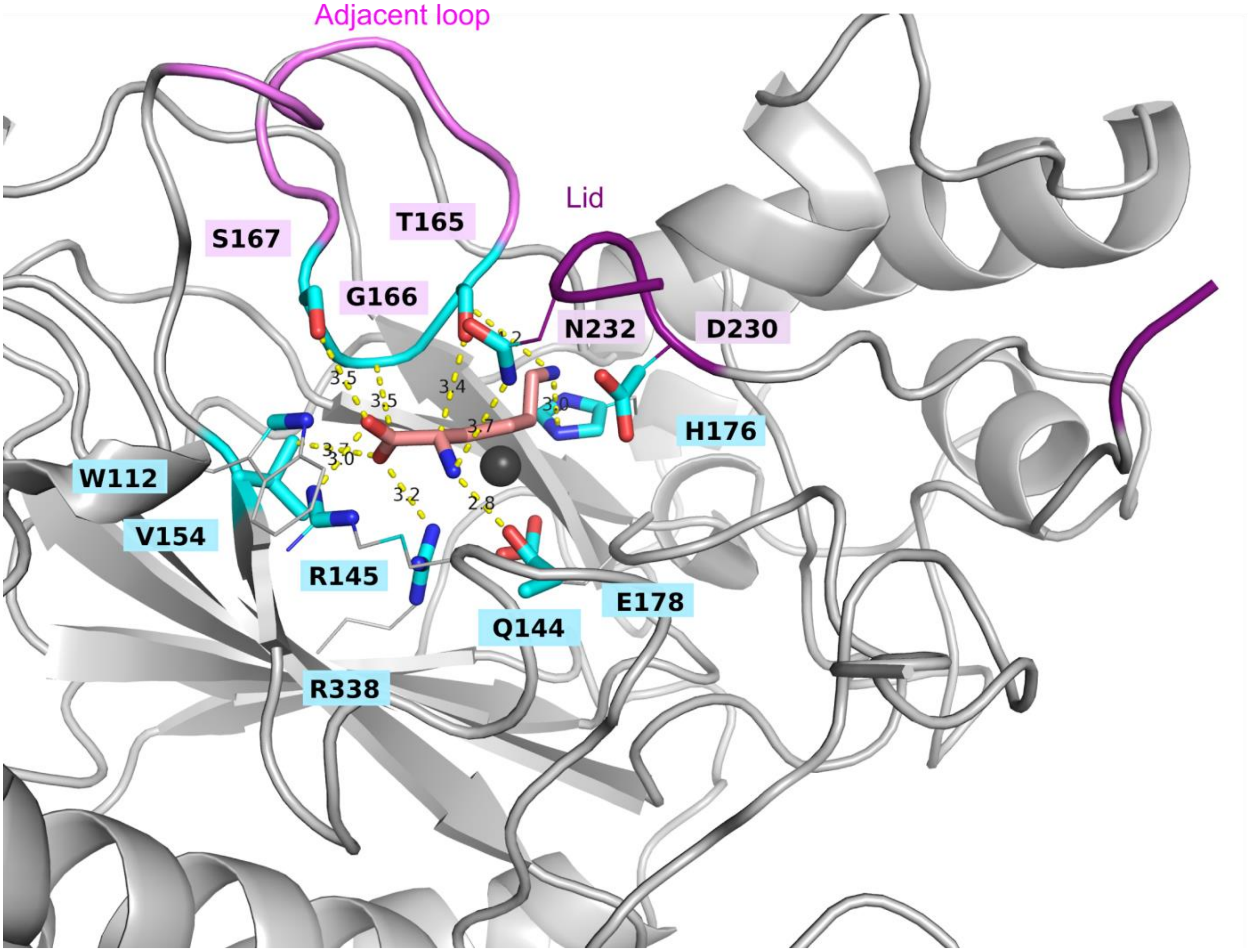

Figure S11 | Interaction between Lysine substrate and KDO5 active site pocket. KDO5 structure is represented by the light gray cartoon. The stick representation of the substrate is salmon-colored. The flexible lid is purple while the adjacent loop is colored pink. Residues of the active site being less than 5 Å from any atoms of the lysine substrate are in stick and colored cyan. Residues names are indicated in rectangles. Possible interactions (electrostatics or van der Waals) between the lysine substrate and the KDO5 are represented by yellow dashed lines. Distances are indicated in Angstrom. The adjacent loop wraps up around one face of the Lysine substrate while the region of the flexible lid covers the top of the active site. The lid also stabilizes the lysine substrate through a potential electrostatic interaction between its α-amino group and Asn232 (3.7 Å).

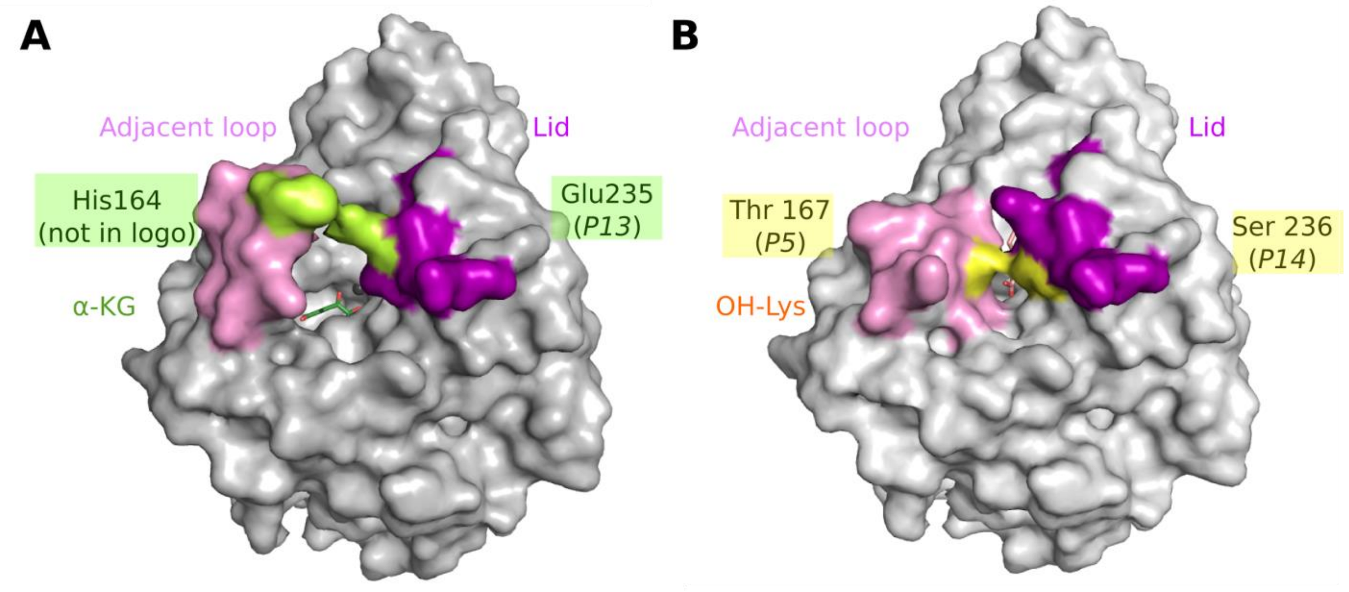

Fig. S12 | Comparison of solvent accessibility between open form (A) and closed form (B) of KDO1. (**A**) Surface representation of KDO1 in complex with α-KG (in green and stick). (**B**) Surface representation of KDO1 in complex with hydroxy-lysine (in salmon and stick) and succinate (hidden by the adjacent loop). Adjacent loop and lid are colored respectively in pink and purple. Residues involved in active site enclosing are colored in lemon and yellow for open and closed form respectively. Their numbering in the sequence and in the logo (in brackets) is indicated.

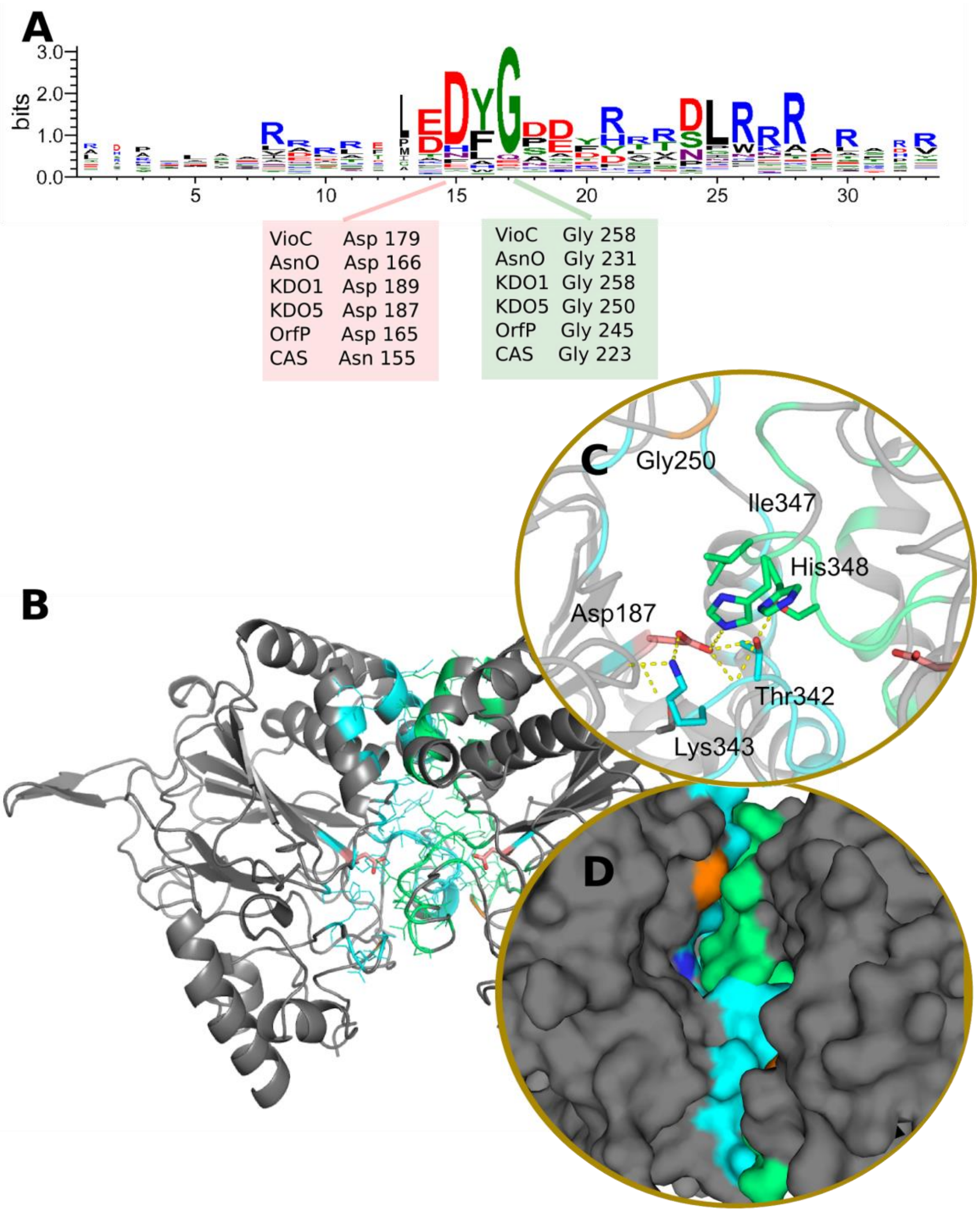

Figure S13 | Representation of dimer interface. (A) Logo representing the conservation of residues involved in the dimeric interface for all members of the CSL family. Asp and Gly, in positions 15 and 17 respectively of the logo, are highly conserved in the family. Corresponding numbering for these two residues in CSL crystal structures is indicated. It should be noted that the interface contains numerous Lys and Arg that give electronegative patches on the contact surface. **(B)** Global overview of the dimers formed by chain A (on left) and chain B (on right) of KDO5. The 33 residues involved in the interface are colored blue for chain A and green for chain B. Conserved Asp for each chain is colored salmon in stick. **(C)** Focus on the highly conserved residue Asp and its interaction with residues of the

other chain. His348 was resolved with two possible rotamers, which highlights its adaptability during the monomers association. **(D)** Surface representation of the dimeric interface. His348 and Ile347 act as a key docking into the lock pocket formed by Asp187.

References

1 Helmetag, V., Samel, S. A., Thomas, M. G., Marahiel, M. A. & Essen, L.-O. Structural basis for the erythro-stereospecificity of the L-arginine oxygenase VioC in viomycin biosynthesis. *FEBS J.* **276**, 3669-3682 (2009).

2 Diederichs, K. & Karplus, P. A. Better models by discarding data? *Acta crystallographica. Section D, Biological crystallography* **69**, 1215-1222 (2013).

3 Kabsch, W. XDS. *Acta Crystallogr. D Biol. Crystallogr.* **66**, 125-132 (2010).

4 Bricogne, G. *et al.* BUSTER version 2.11. 2. *Cambridge, United Kingdom* (2011).

5 Bleuler, S., Laumanns, M., Thiele, L. & Zitzler, E. in *Lecture Notes in Computer Science* 494-508 (2003).

6 Vera, L. & Stura, E. A. Strategies for Protein Cryocrystallography. *Crystal Growth & Design* **14**, 427-435 (2014).

7 Tickle, I. J. *et al.* Staraniso. *Cambridge, United Kingdom: Global Phasing Ltd* (2017).

8 Martinez, S. & Hausinger, R. P. Catalytic Mechanisms of Fe(II)- and 2-Oxoglutarate-dependent Oxygenases. *J. Biol. Chem.* **290**, 20702-20711 (2015).

9 Hanauske-Abel, H. M. & Gunzler, V. A stereochemical concept for the catalytic mechanism of prolylhydroxylase: applicability to classification and design of inhibitors. *Journal of theoretical biology* **94**, 421-455 (1982).

10 Strieker, M., Kopp, F., Mahlert, C., Essen, L.-O. & Marahiel, M. A. Mechanistic and structural basis of stereospecific Cbeta-hydroxylation in calcium-dependent antibiotic, a daptomycin-type lipopeptide. *ACS Chem. Biol.* **2**, 187-196, (2007).

11 Xu, Q. *et al.* Crystal structure of a member of a novel family of dioxygenases (PF10014) reveals a conserved cupin fold and active site. *Proteins* **82**, 164-170 (2014).

12 Lukat, P. *et al.* Biosynthesis of methyl-proline containing griselimycins, natural products with anti-tuberculosis activity. *Chem. Sci.* **8**, 7521-7527 (2017).

13 Hegg, E. L. & Que, L., Jr. The 2-His-1-carboxylate facial triad--an emerging structural motif in mononuclear non-heme iron(II) enzymes. *Eur. J. Biochem.* **250**, 625-629 (1997).

14 Mitchell, A. J. *et al.* Structural basis for halogenation by iron- and 2-oxo-glutarate-dependent enzyme WelO5. *Nat. Chem. Biol.* **12**, 636-640 (2016).

15 Clifton, I. J. *et al.* Structural studies on 2-oxoglutarate oxygenases and related double-stranded β-helix fold proteins. *J. Inorg. Biochem.* **100**, 644-669 (2006).

16 McDonough, M. A., Loenarz, C., Chowdhury, R., Clifton, I. J. & Schofield, C. J. Structural studies on human 2-oxoglutarate dependent oxygenases. *Curr. Opin. Struct. Biol.* **20**, 659-672 (2010).

17 Crooks, G. E., Hon, G., Chandonia, J. M. & Brenner, S. E. WebLogo: a sequence logo generator. *Genome research* **14**, 1188-1190 (2004).

18 Lavery, R. & Sacquin-Mora, S. Protein mechanics: a route from structure to function. *J. Biosci.* **32**, 891-898 (2007).

19 Sacquin-Mora, S. Motions and mechanics: investigating conformational transitions in multi-domain proteins with coarse-grain simulations. *Mol. Simul.* **40**, 229-236 (2013).

20 Sacquin-Mora, S. Bridging Enzymatic Structure Function via Mechanics: A Coarse-Grain Approach. *Methods Enzymol.* **578**, 227-248 (2016).
21 Koketsu, K. *et al.* Refined regio- and stereoselective hydroxylation of L-pipecolic acid by protein engineering of L-proline cis-4-hydroxylase based on the X-ray crystal structure. *ACS Synth. Biol.* **4**, 383-392a (2015).
22 Krojer, T. *et al.* Crystal structure of human aspartate beta-hydroxylase isoform a. doi: 10.2210/pdb5APA/pdb (2011).
